# Biomarker profiling in reef corals of Tonga’s Ha’apai and Vava’u archipelagos

**DOI:** 10.1371/journal.pone.0185857

**Published:** 2017-11-01

**Authors:** Anderson B. Mayfield, Chii-Shiarng Chen, Alexandra C. Dempsey

**Affiliations:** 1 National Museum of Marine Biology and Aquarium, Checheng, Pingtung, Taiwan; 2 Khaled bin Sultan Living Oceans Foundation, Annapolis, Maryland, United States of America; 3 Taiwan Coral Research Center, Checheng, Pingtung, Taiwan; 4 Graduate Institute of Marine Biotechnology, National Dong-Hwa University, Checheng, Pingtung, Taiwan; 5 Department of Marine Biotechnology and Resources, National Sun Yat-Sen University, Kaohsiung, Taiwan; National Taiwan Ocean University, TAIWAN

## Abstract

Given the significant threats towards Earth’s coral reefs, there is an urgent need to document the current physiological condition of the resident organisms, particularly the reef-building scleractinians themselves. Unfortunately, most of the planet’s reefs are understudied, and some have yet to be seen. For instance, the Kingdom of Tonga possesses an extensive reef system, with thousands of hectares of unobserved reefs; little is known about their ecology, nor is there any information on the health of the resident corals. Given such knowledge deficiencies, 59 reefs across three Tongan archipelagos were surveyed herein, and pocilloporid corals were sampled from approximately half of these surveyed sites; 10 molecular-scale response variable were assessed in 88 of the sampled colonies, and 12 colonies were found to be outliers based on employment of a multivariate statistics-based aberrancy detection system. These outliers differed from the statistically normally behaving colonies in having not only higher RNA/DNA ratios but also elevated expression levels of three genes: 1) *Symbiodinium* zinc-induced facilitator-like 1-like, 2) host coral copper-zinc superoxide dismutase, and 3) host green fluorescent protein-like chromoprotein. Outliers were also characterized by significantly higher variation amongst the molecular response variables assessed, and the response variables that contributed most significantly to colonies being delineated as outliers differed between the two predominant reef coral species sampled, *Pocillopora damicornis* and *P*. *acuta*. These closely related species also displayed dissimilar temporal fluctuation patterns in their molecular physiologies, an observation that may have been driven by differences in their feeding strategies. Future works should attempt to determine whether corals displaying statistically aberrant molecular physiology, such as the 12 Tongan outliers identified herein, are indeed characterized by a diminished capacity for acclimating to the rapid changes in their abiotic milieu occurring as a result of global climate change.

## Introduction

**Fig 1:**
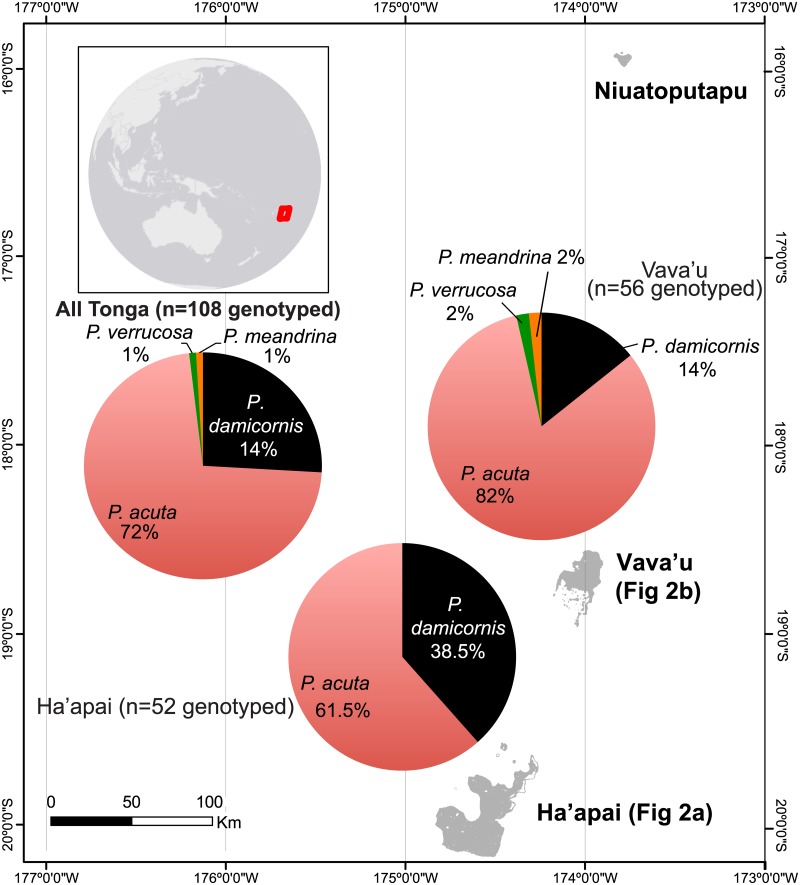
Partial map of the Kingdom of Tonga, including a genetic breakdown of the pocilloporids sampled. Reefs of the island of Tongatapu (the main population center) were not surveyed; therefore, this island, which lies south of Ha’apai, has not been depicted. Pie graphs showing the breakdown of the pocilloporid coral species sampled have been presented for each archipelago in isolation (n = 52 and 56 genotyped colonies for Ha’apai and Vava’u, respectively), as well as pooled across both (n = 108). There was a statistically significant effect of archipelago on host coral genotype proportion (S1 Table; *X*^2^ test, *p*<0.01).

Coral reefs are threatened on a global scale, not only by the rapidly changing climate [1–2], but also by more localized anthropogenic stressors, such as water pollution stemming from poorly regulated coastal development [3–4]. Unfortunately, most of Earth’s reefs are understudied; for instance, the Kingdom of Tonga (Fig 1) possesses a vast reef tract presumably encompassing thousands of hectares of coral reefs, less than 1% of which (typically tourist dive sites) have ever been observed by humankind (the remainder having been inferred from satellite imagery). Furthermore, there is no information on the physiological condition of the framework-building scleractinians in Tonga, and the first surveys of reef coral health in the entire South Pacific region were not even conducted until 2013; a series of physiological and molecular-scale response variables (MSRV) were measured in pocilloporid corals from both French Polynesia’s Austral Islands and the Cook Islands as part of the Khaled bin Sultan Living Oceans Foundation’s (KSLOF) “Global Reef Expedition” (GRE) [5]. Clearly, if there is an interest in monitoring the effects of global climate change and other anthropogenic insults on coral reef and reef coral health, a more concerted survey effort is warranted, particularly in remote, sparsely populated locations. Such isolated reefs could be hypothesized to be more resilient than those of, for instance, Taiwan, which abut some of the world’s most densely populated cities [6].

Although diagnosing disease, bleaching, and death is commonplace in the coral biology field, there are currently no validated approaches for estimating the health of normally pigmented corals aside from simply watching colonies grow and reproduce. However, assessing coral growth and reproductive output is infeasible for most scientists working at remote field sites, where only several weeks at most may be spent. Regarding the former parameter, measuring coral growth *in situ* is time consuming due to the slow rate at which most corals grow; those working off the very research ships that are able to access the most difficult-to-reach reefs are therefore unlikely to have the opportunity to acquire reliable coral growth data. Regarding reproduction, only researchers whose field trips coincide with the target species’ gamete or larval release periods will be able to estimate fecundity. Given these hurdles, researchers have previously attempted to develop a series of molecular biomarkers (*sensu* [7]) for coral health diagnostics (e.g., [8–13]); as doing so would simply require the acquisition of a biopsy from a coral colony at a single time point, biomarker profiling could be conducive for those interested in making inferences about coral health/physiology while working at remote field sites far from marine laboratories.

However, despite a concerted effort by numerous individuals, there are currently no well-validated biomarkers for the determination of coral health [14]. Although a discussion as to why this is the case is beyond the scope of this manuscript, it stems from methodological hurdles (e.g., difficulties in working with an organism comprised of two eukaryotes: the coral host and its endosymbiotic dinoflagellates of the genus *Symbiodinium* [15–17]), as well as gaps in our understanding of the basic biology of reef coral-*Symbiodinium* mutualisms [18–21]. Furthermore, biomarkers validated from manipulative experiments performed in one location may not be the most useful for conspecifics sampled from other locations; a gene or protein that is up-regulated in response to elevated temperature in corals from Taiwan, for instance, may not show a similar trend in conspecifics from elsewhere in the Indo-Pacific due to differing environmental histories. In the absence of biomarker expression/concentration/activity data from corals sampled prior to the Industrial Revolution (at which point coral health was likely influenced by humankind to a significantly lesser extent), it is unclear what is actually a normal or “healthy” coral phenotype; indeed, few (if any) of Earth’s reefs could currently be said to be in a pristine state.

Despite issues with employing molecular biomarkers, such as gene mRNAs, to assign a level of health or stress to a sampled reef coral, it is nevertheless possible that a battery of such genes could be used to calculate a baseline molecular phenotype for corals of a particular habitat/region. Not only would the ensuing dataset serve as a comparison/reference for future monitoring endeavors, but the data could also be used to demark corals displaying statistically aberrant cellular behavior at the time of sampling. It should be noted here that “aberrant” simply refers to deviation from a local norm and does not reflect a level of health. Although such aberrantly behaving colonies may ultimately be found to be those that are of compromised resilience, they could, alternatively, simply be characterized by the greatest degree phenotypic plasticity; this property could actually *better* enable them to cope with future changes in their environment.

**Fig 2:**
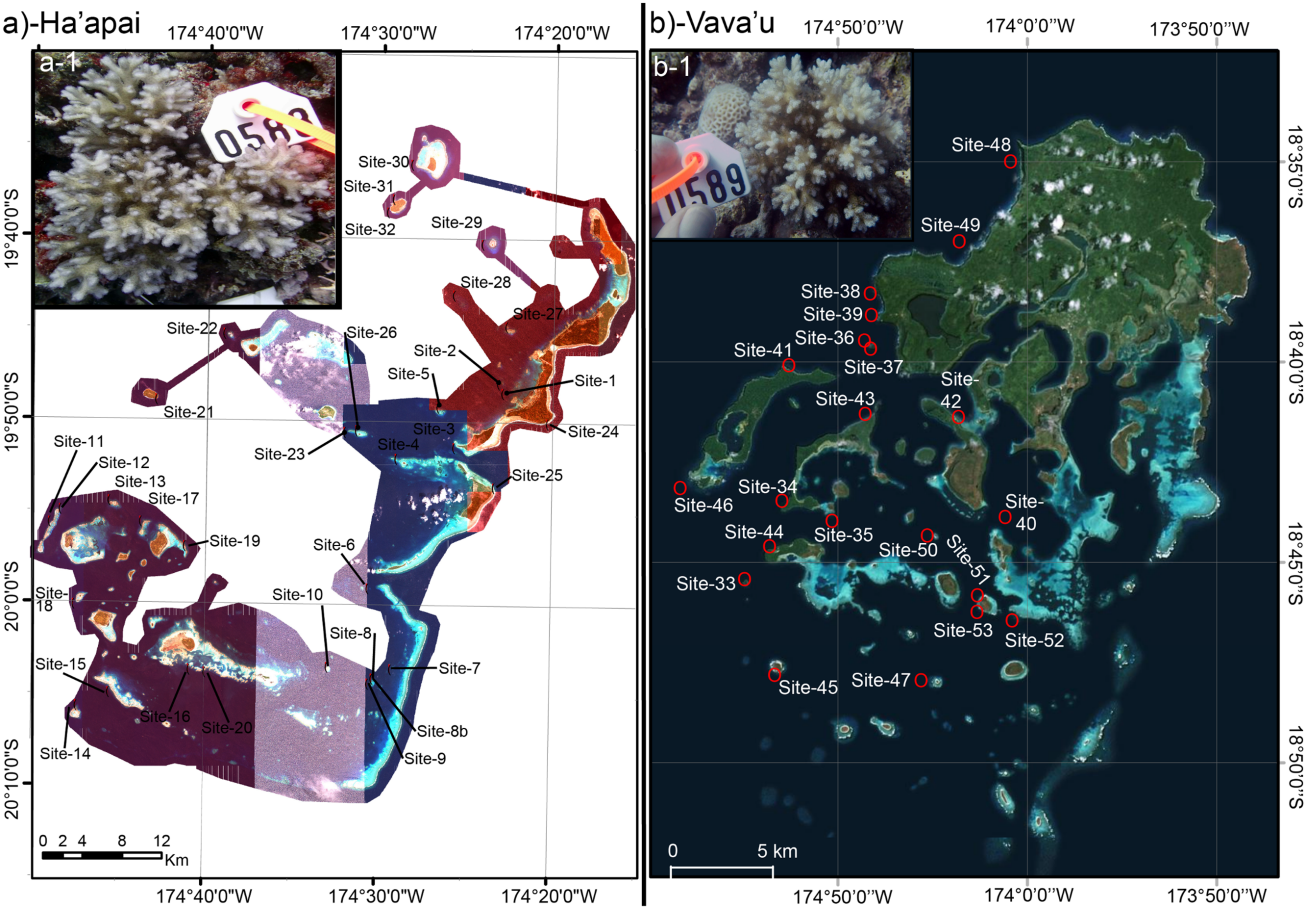
Maps of the two Tongan archipelagos from which pocilloporid corals were sampled: Ha’apai (a) and Vava’u (b). The site codes correspond to those found in Tables 1 and 2, respectively, albeit excluding the “TOHA” and “TOVA” prefixes, respectively. Insets in (a) and (b) show representative *Pocillopora damicornis* (a-1; genotype α) and *P*. *acuta* (b-1; genotype β) colonies, respectively, and the length of the white tag (the scaling object) in each is 4.7 cm.

Given the needs to 1) survey historically understudied reefs and 2) identify corals displaying statistically aberrant behavior with respect to a number of MSRV (notably gene mRNAs), 59 Tongan coral reefs were surveyed, and the model coral *Pocillopora damicornis* (Fig 2a-1; [22–24]) or, alternatively, its closely related sister species *P*. *acuta* (Fig 2b-1), were sampled from half of these reefs in order to attempt to remedy the latter knowledge dearth. Although these two species appear virtually identical *in situ*, we hypothesized that they might differ physiologically since they were previously found to occupy different niches elsewhere in the South Pacific [5]. It was also hypothesized that 1) a multivariate statistical approach (MSA) could be used to identify outliers and 2) the distribution of such outliers would differ across environmental gradients (e.g., average live coral cover [ALCC; %] levels, depth, etc.). Specifically, outliers were hypothesized to be more abundant in marginalized reefs characterized by low ALCC. The baseline molecular, physiological, and ecological data generated were envisioned to collectively serve as a comparative resource for those looking to track the condition of South Pacific coral reefs in this era of rapidly changing climate.

**Table 1:** Ha’apai site information. Of the 32 sites surveyed at Ha’apai, the target coral species *Pocillopora damicornis*, or, alternatively, its closely related sister species *P*. *acuta*, was observed at and sampled from 15, and the data in this table are from these 15 sites only. In total 54 pocilloporid coral colonies were sampled across these 15 sites, and neither species was observed at the following 10 sites: TOHA03, 21, 22, 24, 25, 27, and 29–32. For the following seven sites, *P*. *damicornis/P*. *acuta* may have been present, but neither was sampled: TOHA02, 6, 11–12, 16–17, and 20. All reefs were emergent unless noted otherwise. For the environmental data for all 32 surveyed sites, please see S1 Data. The average temperatures (temp.), salinities, and live coral cover (ALCC) percentages (±std. dev.) reflect the means across the 15 sites from which corals were sampled (not all 32 surveyed sites). The 10 samples highlighted in bold were *not* processed for all molecular-scale response variables (MSRV).

Site	Exposure	Reef type	Reef zone	Latitude	Longi-tude	Date (2013)	Temp. (°C)	Salinity (unit-less)	ALCC (%)	#corals analyzed/ #collected (sample ID#)	*P*. *damicornis* (PD)*/P*. *acuta* (PA) present
TOHA01	protected	patch	lagoonal	-19.8064	-174.3803	Sept. 11	23.8	35.6	30±21	6/6 (#1–6)	PD (n = 1) & PA (n = 5)
TOHA04	intermediate	patch	lagoonal	-19.8668	-174.4824	Sept. 12	24.3	35.3	24±5.0	2/2 (#7–8)	PD (n = 1) & PA (n = 1)
TOHA05	intermediate	patch	lagoonal	-19.8198	-174.4426	Sept. 12	24.3	35.4	33±9.7	0/1 (#**9**)	unknown^a^
TOHA07	protected	patch^b^	lagoonal	-20.0581	-174.4856	Sept. 13	24.2	35.5	17±4.7	2/3 (#**10**, 11–12)	PD only (n = 3)
TOHA08	protected	patch	lagoonal	-20.0668	-174.5032	Sept. 13	24.3	35.2	24±5.1	5/5 (#13–17)	PD (n = 2) & PA (n = 3)
TOHA09	protected	patch	lagoonal	-20.0714	-174.5074	Sept. 14	24.1	35.4	25±5.9	6/8 (#**18**, 19, **20**, 21–25)	PD (n = 5)^a^ & PA (n = 2)
TOHA10	protected	patch	lagoonal	-20.0566	-174.5468	Sept. 14	24.4	35.3	40±4.7	3/3 (#26–28)	PA only (n = 3)
TOHA13	intermediate	patch	lagoonal	-19.9071	-174.7584	Sept. 15	24.4	35.3	24±11	2/2 (#29–30)	PA only (n = 2)
TOHA14	intermediate	barrier	forereef	-20.0938	-174.7885	Sept. 16	24.2	35.5	42±11	0/1 (#**31**)	PA only (n = 1)
TOHA15	intermediate	fringing	forereef	-20.0817	-174.7574	Sept. 16	24.3	35.4	41±7.7	3/4 (#32–33, **34**–**35**)	PD (n = 1) & PA (n = 3)
TOHA18	intermediate	barrier	forereef	-20.0016	-174.7918	Sept. 17	24.5	35.4	51±19	1/1 (#36)	PA only (n = 1)
TOHA19	intermediate	fringing	forereef	-19.9478	-174.6852	Sept. 17	25.0	35.4	26±8.8	5/7 (#37–39, **40–41**, 42–43)	PD (n = 1) & PA (n = 6)
TOHA23	protected	patch	lagoonal	-19.8429	-174.5321	Sept. 18	25.6	35.3	33±12	5/5 (#44–48)	PA only (n = 5)
TOHA26	intermediate	fringing	forereef	-19.8413	-174.5209	Sept. 19	25.5	35.3	29±9.9	2/3 (#49–**51**)	PD only (n = 3)
TOHA28	protected	patch	lagoonal	-19.7176	-174.4283	Sept. 20	25.8	35.3	30±9.5	3/3 (#52–54)	PD only (n = 3)
				**Ha’apai avg.±std. dev**.			24.6±0.6	35.4±0.1	31±8.9		20 PD (38.5%)
			Total # analyzed for MSRV/total # genotyped							44/52	32 PA (61.5%)

^a^Certain samples at site were not genotyped, so other pocilloporid species may have been present.

^b^submergent reef.

**Table 2:** Vava’u site information. Of the 21 sites surveyed at Vava’u, the target coral species *Pocillopora damicornis*, or, alternatively, its closely related sister species *P*. *acuta*, was sampled from 10, and the data in this table are from these sites only (in addition to a “snorkel site” where only several minutes were spent). In total 61 pocilloporid corals were sampled, though neither species was observed at the following eight sites: TOVA36-38, 45–46, 48–50. For the following three sites, *P*. *damicornis/P*. *acuta* may have been present, but no colonies were sampled: TOVA41-43. For the environmental data for all 21 surveyed sites, please see S1 Data. All reefs were emergent unless noted otherwise. The average temperatures (temp.), salinities, and live coral cover (ALCC) percentages (±std. dev.) in the “Vava’u avg.±std. dev.” and “Tonga avg.±std. dev.” rows reflect the means across the 10 Vava’u and 25 Tongan (15 Ha’apai+10 Vava’u) sites from which corals were sampled (excluding the snorkel site at Vava’u, where none of these parameters were measured), respectively, and the temp. was significantly higher at Vava’u than at Ha’apai (student’s *t*-test, *p*<0.05). The 17 samples highlighted in bold were not processed for all molecular-scale response variables (MSRV). PM = *P*. *meandrina*. PV = *P*. *verrucosa*. ND = not determined.

Site	Exposure	Reef type	Reef zone	Latitude	Longi-tude	Date (2013)	Temp. (°C)	Salinity (unit-less)	ALCC (%)	#corals analyzed/ #collected (sample ID#)	*P*. *damicornis* (PD)/*P*. *acuta* (PA) present
TOVA33	protected	patch	lagoonal	-18.7570	-174.1228	Sept. 22	26.0	35.3	33±14	1/1 (#55)	PM only (n = 1)
TOVA34	intermediate	fringing	forereef	-18.7244	-174.1064	Sept. 22	26.4	35.3	50±5.6	4/7 (#56–57, **58**–59, **60**, 61, **62**)	PA only (n = 7)
TOVA35	protected	patch	lagoonal	-18.7328	-174.0845	Sept. 22 Sept. 25	26.0	35.3	41±6.9	7/11 (#63, **64, 65, 66**, 77–78, **79**, 80–83)	PD (n = 3) & PA (n = 7)^a^
TOVA39	intermediate	fringing	forereef	-18.6470	-174.0670	Sept. 24	26.5	35.2	16±15	1/1 (#67)	PV (n = 1)
Snorkel site (near “Japanese Coral Gardens” dive site)						Sept. 24	ND	ND	ND	1/1 (#68)	PA only (n = 1)
TOVA40	protected	patch^b^	lagoonal	-18.7308	-174.0098	Sept. 24	25.8	35.4	24±3.0	6/8 (#69–73, **74**, 75, **76)**	PA only (n = 8)
TOVA44	protected	fringing	forereef	-18.7434	-174.1119	Sept. 25	26.7	35.2	51±22	6/6 (#84–89)	PD (n = 2) & PA (n = 4)
TOVA47	intermediate	fringing	lagoonal	-18.7989	-174.0451	Sept. 26	26.3	35.3	44±27	6/8 (#**90–91**, 92–97)	PD (n = 1) & PA (n = 7)
TOVA51	protected	fring-ing^b^	backreef	-18.7634	-174.0206	Sept. 27	26.4	35.3	35±7.9	4/8 (#98, **99**, 100–**101**, 102, **103**, 104–**105)**	PA only (n = 6)^a^
TOVA52	protected	patch^b^	lagoonal	-18.7740	-174.0054	Sept. 28	26.2	35.3	47±24	1/3 (#106, **107**, **108**)	PA only (n = 1)^a^
TOVA53	protected	barrier	backreef	-18.7705	-174.0207	Sept. 28	26.1	35.3	38±28	7/7 (#109–115)	PD (n = 2) & PA (n = 5)
				**Vava’u avg.±std. dev**.			26.2±0.3	35.3±0.1	38±11		8 PD (14%), 1 PM (2%), 1 PV (2%), 46 PA (82%)
		Total # analyzed for MSRV/total # genotyped (Vava’u)								44/56	
				**Tonga avg. ± std. dev**.			25.2±0.97	35.3±0.09	34±10		
		Total # analyzed for MSRV/total # genotyped (Ha’apai+Vava’u)								88/108	

^a^Certain samples at site were not genotyped, so other pocilloporid species may have been present.

^b^submergent reef.

## Materials and methods

### Field surveys and sample collection

In September 2013, the KSLOF research vessel *M*.*Y*. *Golden Shadow* traversed a significant portion of the Kingdom of Tonga’s territorial waters (Fig 1) upon invitation from the King. Remote sensing, field surveys, and pocilloporid coral collection were carried out as described previously [5], and certain details have been reiterated in the S1 File. Briefly, sites were chosen by analysis of satellite data in conjunction with visual observations from seaplane flyovers. Benthic maps were created (*sensu* [25]), and shallow-water (5–30 m) coral reef surveys were conducted to document the dominant benthic organisms and substrate types. In total, 59 sites were surveyed over three weeks, and three major regions were visited: the Ha’apai Archipelago (Table 1 and Fig 2a; n = 32 sites surveyed over 11 days), the Vava’u Archipelago (Table 2 and Fig 2b; n = 21 sites surveyed over 7 days), and Niuatoputapu Volcano (n = 6 sites surveyed over 2 days); however, since the target species were not present at the latter region, the site descriptions and ecological data from Niuatoputapu are discussed/presented only in the S1 File and S1 Data, respectively.

As mentioned above, the target coral for biomarker profiling was the α genotype of *P*. *damicornis* [26], though when it was not present, the morphologically similar congeneric *P*. *acuta* (genotype β) was instead sampled as in a published reference [5]; briefly, corals were sampled across a range of depths (typically 5 to 30 m), habitat types (e.g., forereefs, lagoons, patch reefs, etc.), and environmental gradients (e.g., temperatures) in order to understand the relationship between environment and coral physiology (described in detail below). Coral collection was approved by Tonga’s 1) King, 2) Ministry of Lands, Environment, Climate Change, and Natural Resources (MLECCNR), and 3) Ministry of Agriculture, Forests, and Fisheries (MAFF); ministers from the latter two departments were onboard during the mission and assisted in research efforts.

Of the 115 sampled colonies, 108 were genotyped (discussed below), and all but two were found to be the target species; one *P*. *verrucosa* colony (#67 from Vava’u) was inadvertently sampled, as was one *P*. *meandrina* colony (#55 from Vava’u). Although these two samples were processed for the 10 MSRV discussed below, they were generally excluded from the data analysis. While *in situ*, it was noted whether the polyps of the sampled colonies were expanded/ extended (i.e., outside of their corallites), and colonies were scored as either “polyps extended” (considered as such even if only a small portion of the polyps were extended) or “polyps retracted.” The colonies were then photographed with a scaling object; both maximum (max.) colony length (cm) and planar surface area (SA; cm^2^) were later determined *in silico* via image analysis with ImageJ (National Institutes of Health, USA). Color was estimated *in situ* and ranked on a scale of 1 to 4 (1 = normal, 2 = pale, 3 = very pale, and 4 = bleached), though due to subjective nature of this parameter, it was generally excluded from most MSA (with the exception of the multivariate ANOVAs [MANOVA] of the S1 and S2 Tables). Similarly, the two size parameters were excluded from most MSA and the outlier assignment analysis (both described below) since colony size was not predicted to be useful in assigning a level of aberrancy to a colony. The polyp expansion data were categorical and so were not incorporated into the MSA or outlier assignment analysis.

### Nucleic acid extractions and MSRV

From the 115 sampled colonies, both RNAs and DNAs were extracted with TRIzol^®^ (Life Technologies) as described previously [5]. Only samples for which both high quality RNA (two distinct bands [28s and 18s rRNA] seen in the ethidium bromide-stained agarose [1%] gel, 260/280 ratio of 1.9–2.1, and 260/230 ratio >2) and DNA (a single high molecular weight band seen in the ethidium bromide-stained agarose [1%] gel, 260/280 ratio of 1.7–1.9, and 260/230 ratio>2) were processed for all 10 MSRV and considered in the statistical analyses described below. First, an RNA/DNA ratio was calculated for each sample to serve as a proxy for total gene expression (MSRV#1). Then, 108 and 88 colonies were genotyped and analyzed for the remaining nine MSRV described below, respectively; all of the latter 88 samples were genotyped. Four *Symbiodinium* genes (MSRV#2–5) and four host coral genes (MSRV#6–9) were targeted for mRNA expression analysis after conversion of RNA to cDNA as described previously [27]. The former four (Table 3) included the photosynthesis gene ribulose-1,5-bisphosphate carboxylase/oxygenase large subunit (*rbcL*), the metabolism gene zinc-induced facilitator-like 1-like (*zifl1l*; known to be down-regulated at high temperature in *Symbiodinium* populations within *P*. *damicornis* [24]), and two genes encoding proteins involved in the cellular stress response: heat shock protein 90 (*hsp90*) and ubiquitin ligase (*ubiq-lig*; [30]). The host target genes were the metabolism gene carbonic anhydrase (*ca*), the cell adhesion gene *lectin*, the stress gene copper-zinc superoxide dismutase (*cu-zn-sod*), and the light absorbing gene green fluorescent protein-like chromoprotein (*gfp-cp*). Please see Table 3 for hypotheses regarding gene expression behavior for each of the eight mRNAs targeted. Real-time PCR (qPCR)-based gene expression quantification of these eight targets was carried out as described previously [31].

**Table 3:** Molecular-scale response variables (MSRV). Although 12 quantitative (10 MSRV+2 size parameters) and two categorical (color and polyp expansion) response variables were assessed for each of 88 of the 115 pocilloporid colonies sampled herein, only the 10 MSRV were hypothesized to be useful in assigning a level of aberrancy to the sampled colonies. Statistically significant findings (student’s *t*-test of outlier vs. non-outlier, *p*<0.05) have been underlined. One *Symbiodinium* (*zifl1l*) and two host coral mRNAs (*cu-zn-sod* and *gfp-cp*) were found to be significantly higher in outliers than non-outliers in both the Fiji and Tonga datasets.

MSRV	Abbrevia-tion	Proxy/Cellular Function	Hypothesis	Observed result (Tonga)	Results from Fiji^h^
**Biological composition** (n = 2)		**PROXY**			
RNA/DNA ratio^a^	RNA/DNA	total transcription	Too high or too low levels indicative of aberrant behavior	outlier>non-outlier	outlier = non-outlier
*Symbiodinium* genome copy proportion^b^	Sym GCP	Sym density	Too high or too low levels indicative of aberrant behavior	outlier = non-outlier	outlier<non-outlier
**Sym mRNA expression** (n = 4)		**CELLULAR FUNCTION**			
ribulose-1,5 bisphosphate carboxylase/oxygenase^c^	Sym *rbcL*	photosynthesis	Too low levels indicative of aberrant behavior	outlier = non-outlier	outlier>non-outlier
zinc-induced facilitator-like 1-like^d^	Sym *zifl1l*	zinc transport	Too high or too low levels indicative of aberrant behavior	outlier>non-outlier	outlier>non-outlier
heat shock protein 90^d^	Sym *hsp90*	stress response	Too high levels indicative of aberrant behavior	outlier = non-outlier	outlier>non-outlier
ubiquitin ligase^d^	Sym *ubiq-lig*	stress response^g^	Too high levels indicative of aberrant behavior	outlier = non-outlier	outlier>non-outlier
**Host coral mRNA expression** (n = 4)					
carbonic anhydrase^d^	host *ca*	carbon metabolism	Too low levels indicative of aberrant behavior	outlier>non-outlier	outlier = non-outlier
lectin^e^	host *lectin*	cell adhesion	Too low levels indicative of aberrant behavior	outlier = non-outlier	outlier = non-outlier
copper-zinc superoxide dismutase^d^	host *cu-zn-sod*	stress response	Too high levels indicative of aberrant behavior	outlier>non-outlier	outlier>non-outlier
green fluorescent protein-like chromoprotein^f^	host *gfp-cp*	light absorption	Too high or too low levels indicative of aberrant behavior^i^	outlier>non-outlier	outlier>non-outlier

^a^[27].

^b^[17].

^c^[28].

^d^[24].

^e^[12].

^f^[29].

^g^[30].

^h^[31].

^i^See Discussion for more details.

From the DNAs co-extracted from the same biopsies from which RNAs were isolated, a genome copy proportion (GCP) was calculated to serve as a proxy for *Symbiodinium* density (MSRV#10); in addition to the recovery of an exogenous Solaris™ RNA spike (discussed in detail in a prior work [32]), host and *Symbiodinium* gene expression data were normalized to the host and *Symbiodinium* GCP, respectively. This parameter controls for differences in the host/*Symbiodinium* biomass ratio between samples [17], which can vary greatly due to, for instance, bleaching [33], or, alternatively, failure to effectively extract nucleic acids from *Symbiodinium* cells within certain coral biopsies.

In addition to *Symbiodinium* density estimates, the co-extracted DNAs were also used for clade-level *Symbiodinium* genotyping with the primers and qPCR assays of Correa et al. [34]. When a threshold cycle (Ct) value lower than 35 was documented for a particular assay, the sample was deemed positive for the respective clade. Finally, the DNAs were also used to genotype the host corals by PCR amplifying a portion of the mitochondrial genome encompassing the 3’ end of the ATP synthase (subunit 6) gene and the 5’ end of the mitochondrial control region (also known as the mitochondrial open reading frame [mORF]). Details of the genotyping protocol and consequent sequence analysis can be found in Mayfield et al. [5]. Samples were assigned to one of four species (Fig 1).

### Overview of statistical analyses

Since both environmental and physiological data were acquired during the mission, there were two major analytical goals. First, a variety of statistical analyses were utilized to attempt to understand the relationship between environment and coral physiology. Then, MSA were used to identify outliers in the dataset to determine whether outlier frequency varied across environmental gradients. Previous studies have employed a combination of univariate and multivariate statistical approaches to analyze coral reef ecological data in isolation, reef coral physiological data in isolation, and both environmental and molecular physiological data in tandem [31–32]; readers are referred to these works for a more detailed treatise on the rationale for using the suite of statistical approaches employed herein. Briefly, both univariate and multivariate statistical approaches were exploited because it was hypothesized that statistically significant, biologically meaningful differences in molecular physiology might not be identified using only the former strategy. For instance, MANOVA can identify combinations of response variables that best partition samples from different environments, even when the underlying response variables show no environmental variation when analyzed in isolation with ANOVA [35]. JMP (ver. 12.0.1) was used for all statistical analyses outlined below except for multi-dimensional scaling (MDS) and analysis of similarity (ANOSIM).

First, univariate statistical approaches were used to test for the effects of the environmental factors measured on the response variables quantified in the coral samples within a primarily ANOVA-based framework. MANOVAs employing a canonical correlation analysis (CCA) algorithm were also used to model the effects of environment on coral physiology. Next, both principal components analysis (PCA) and MDS were used to uncover variation in the dataset and similarity amongst samples, respectively.

Outliers were identified using a dual-calculation approach, and both methods are described in greater detail below and in a prior reference [31]. First, the Mahalanobis distance, which is essentially the multivariate equivalent of the standard deviation, must have been greater than the upper control limit (UCL) calculated by JMP were a sampled to be considered an outlier. Although the Mahalanobis distance is the most statistically rigorous means of detecting outliers, it does take the correlation structure into consideration. This means that, in certain cases, samples may have very “typical” expression levels of certain genes, yet be flagged as outliers due instead to their falling outside of the correlation structure of the dataset. For this reason, we instated a second requirement for demarcating a sample as an outlier: the sample had to have at least one response variable that was over 2.5 standard deviations above the mean (*z*-score>2.5). In certain cases, the sample may have had a “heat map score” of 1, but it would only be considered an outlier were its Mahalanobis distance above the UCL. Finally, we looked to check that these outliers were the most distant and distinct points in the PCA and MDS plots. Although these ordination techniques use completely different algorithms, they have tended to support the strictly quantitative outlier detection assignments described above in prior works [31–32]. This conservative system of mathematical “checks and balances” ensured that statistically normal samples were not inadvertently misclassified as outliers. Following the outlier assignment exercise, we used a series of approaches to determine which response variables were most important in partitioning outliers from non-aberrantly behaving coral colonies. All such approaches are discussed in greater detail below.

### Univariate statistical analyses

As a preliminary analytical step, we tested the effect of environment (14 parameters) on 1) coral molecular physiology (12 response variables; using ANOVA) and 2) host coral, outlier, and colony polyp expansion frequency (using *X*^2^ tests). The 14 environmental parameters included 1) archipelago (n = 2: Ha’apai vs. Vava’u), 2) site (n = 25 reefs from which pocilloporid corals were sampled; Tables 1 and 2), 3) exposure (exposed [typically windward], protected [typically leeward or lagoonal], or intermediate [neither exposed nor protected]), 4) reef zone (forereef, backreef, or lagoon), 5) reef type (barrier, patch, or fringing reef), 6) collection time (n = 3 categorical groupings: <10:00, 10:00–14:00, or >14:00), 7) collection date (n = 15 sampling days), 8) collection depth (n = 7 categorical groupings: <5, 5–10, 10–15, 15–20, 20–25, 25–30, or >30 m), 9) site temperature (n = 4 categorical groupings: 23–24, 24–25, 25–26, or 26–27°C), 10) site salinity (35.2, 35.3, 35.4, 35.5, or 35.6), 11) ALCC (n = 5 categorical groupings: 10–20, 20–30, 30–40, 40–50, or >50%), 12) host species (n = 4; see Fig 1.), 13) colony color (see classifications above.), and 14) *Symbiodinium* assemblage (clade C only or clades A+C [no samples possessed *Symbiodinium* of any other clades.]). Although the latter three variables are not environmental parameters, they were nevertheless hypothesized to influence coral physiology.

Host genotype, outlier, and polyp expansion frequency were also tested as response variables, and such frequency data were analyzed with contingency table-based *X*^2^ tests. Since host frequency, outlier frequency, and polyp expansion frequency were assessed across 13, 14, and 14 environmental parameters, respectively (see S1 Table.), Bonferroni adjustments of 3.6, 3.7, and 3.7, respectively, were made to the *a priori*-chosen α level of 0.05, resulting in a modified α level of 0.013. All other response variables, including max. colony length, planar SA, and the 10 MSRV (see Table 3.) were analyzed across the 14 environmental parameters with 1-way ANOVAs after confirming both normality (Shapiro-Wilk *W* test, *p*>0.05) and homogeneity of variance (Levene’s test, *p*>0.05) of the data. Transformations were conducted when the latter two conditions were not met.

As the identification of systematic outliers (discussed in detail below) was amongst the major analytical goals of this work, outliers were *not* excluded from ANOVA-based analyses; although their inclusion decreased our chances of uncovering significant environmental effects on coral physiology, we justify this inclusion because the associated high variation between coral colonies appears to be common on tropical coral reefs based on prior studies [31–32]; in other words, high inter-sample variation did not simply stem from measurement errors, which we attempted to avoid by using only those 88 samples of the 115 total for which both high quality RNA *and* DNA were extracted (i.e., some samples were excluded from analysis since they were likely to have introduced measurement-derived error into the analysis.). We therefore advocate that other coral ecophysiologists include such outliers in their analyses unless aberrant behavior can be tracked to a measurement error (e.g., the extraction of low-quality RNA that consistently performed poorly in qPCR). By excluding such outliers, one does indeed have a better chance of uncovering environmental effects on coral physiology; however, important information about the population’s phenotypic and physiological variability is lost. Such properties may be important for gauging a coral population’s capacity to acclimate to future environmental change.

Because 168 ANOVAs were performed, a Bonferroni adjustment of 13 was made to the *a priori*-chosen α level of 0.05, resulting in a modified α of 0.004. Similarly, a 13 environmental parameter (the same as above, though excluding host species) x 12 response variable 1-way ANOVA matrix was also generated individually for each of the two predominant species sampled: *P*. *damicorni*s and *P*. *acuta*. For these species-specific comparisons, the *a priori*-chosen α level of 0.05 was instead divided by the square root of 156 to generate the Bonferroni-adjusted α level (also 0.004). In general, only results for which 1) there was an ANOVA-derived difference (*p*<0.004) *and* 2) Tukey’s *post-hoc* differences were documented between individual means (*p*<0.05) were considered to be biologically meaningful. Only the former of these two conditions was generally met for tests of site and date, and, for that reason, we do not discuss such spatio-temporal differences in any detail throughout the manuscript. Finally, for each of the 12 response variables, the coefficient of variation was calculated in addition to the mean and standard deviation to quantify the overall variation between the sampled colonies.

### MSA

Although a Bonferroni adjustment may suffice in controlling for type I statistical errors, MSA can uncover relationships amongst response variables across environments at a lower false positive error rate. Several MSA were taken herein to understand the relationship between environment and coral physiology; all data were converted to *z*-scores prior to MSA to account for the response variables having different units and scales. First, MANOVA was performed to determine the effect of each of the 14 environmental parameters on the multivariate mean centroid calculated across the 10 MSRV. The two size-based parameters were excluded from this analysis (and all those henceforth). Briefly, MANOVA tests whether vectors of means (rather than simply individual means) of different samples are from the same distribution. JMP’s CCA algorithm was used in conjunction with MANOVA to model environmental effects for data from both host coral species; however, since *P*. *damicornis* and *P*. *acuta* showed distinct molecular physiological differences from each other over time, separate MANOVAs were then performed for each species across the 13 environmental parameters (the 14 aforementioned minus “host”).

PCA was next performed with the 10 MSRV to determine the combinations of parameters that best accounted for variation in the dataset (*sensu* [20]). As an alternate, ordination-based means of visualizing the dataset in multiple dimensions, PRIMER (ver. 5) was used to construct a Bray-Curtis similarity matrix (S1 Data), and an MDS plot based off of this matrix was created using data from the 86 samples analyzed for all MSRV (2 of the 88 samples analyzed for all 10 MSRV were omitted from the MSA due to their being non-target species [colonies #55 and 67].). PRIMER’s ANOSIM function was used to test for the effects of the 14 environmental parameters on separation of samples within the dataspace generated. Upon uncovering a statistically significant ANOSIM finding (*p*<0.013 [see explanation of the Bonferroni adjustment above.], the MDS plot was consulted to determine whether the difference was obvious to the naked eye; only statistically significant *and* visually compelling environmental differences in coral physiology have been discussed in the text. For a more detailed explanation of MANOVA+PCA and MDS+ANOSIM, readers are referred to a classic biometry text [36] and Clarke and Warwick [37], respectively. It was hypothesized that both PCA and MDS could be used corroborate the outliers identified (described below).

### Outlier analysis

As described above, Mahalanobis distances were calculated using the 10 MSRV only. Samples characterized by distance values above the UCL of ~4 calculated by JMP were considered to be “Mahalanobis distance outliers.” Then, a heat map was constructed with JMP, and samples with *z*-scores >2.5 for a certain response variable were given a score of 1. For instance, if a sample had a *z-*score of 3 for 1 of the 10 MSRV, it would be given a heat map score of 1. When a sample was considered a Mahalanobis distance outlier *and* had a heat map score of 1 or greater, it was considered an outlier. This outlier assignment schematic approximates that of Mayfield et al. [31–32] though is somewhat more stringent.

Several measures were taken to understand the response variable(s) that contributed most to a sample being deemed an outlier. First, JMP’s predictor screening function was used to rank the 10 MSRV in order of their contribution to the cumulative difference between the 12 outliers uncovered and all other samples (n = 74). Then, a “variability index” was calculated by taking the standard deviation across the *z*-scores of all 10 MSRV for each sample. It was hypothesized that those samples with high Mahalanobis distances (i.e., the outliers) would also demonstrate more variability between the individual response variables assessed, particularly gene expression. This variability index was regressed against the Mahalanobis distance, and the significance of the correlation was tested with a linear regression *t*-test. Differences in certain response variables between outliers and non-outliers were compared across both *P*. *damicornis* and *P*. *acuta* with 2-way ANOVA (outlier status vs. species), and individual mean differences between the four interaction groups were tested with Tukey’s honestly significant difference (HSD) tests (*p*<0.05). Next, MANOVA+CCA was performed using JMP’s “discriminant analysis” function to determine the response variables that best led to the separation of samples characterized by different heat map scores (which ranged from 0 [non-outliers] to 3 [extreme outliers]). This CCA was repeated individually for *P*. *damicornis* and *P*. *acuta*. An α level of 0.05 was established *a priori* for all such MSA, though a Bonferroni adjustment of 3.6 was made to the α levels of the MANOVAs of the S1 and S2 Tables since 13–14 comparisons were made; this resulted in an adjusted α of 0.013.

## Results

### Coral reef ecology

As this represents the first in-depth survey of coral reefs of Tonga, detailed site descriptions have been provided for the majority of the 59 surveyed sites in the S1 File. All images of the samples colonies, as well as their surrounding habitats (typically 1-30-m depth), were taken by ABM, and they can be found on the following website: coralreefdiagnostics.com. At least one “macro” image of the polyps has been included for each colony (n = 115) on this same website. Additionally, all habitat and sampled coral colony images can be downloaded from Dryad (doi:10.5061/dryad.6vj6n). Interactive maps of the surveyed reefs were generated, in part, by ACD (KSLOF), and they can be accessed and manipulated freely on the following website: http://maps.lof.org/lof. The ALCC and other environmental data from sites from which pocilloporid corals were sampled can be found in Tables 1 and 2 for Ha’apai and Vava’u, respectively; for a more detailed treatise of the coral cover data, please see the S1 File. To peruse all environmental data, and not just those from the sites from which corals were collected, please see S1 Data. Briefly, ALCC of Tonga’s reefs was 31±13% (std. dev. for this and all error terms henceforth), and it did not vary significantly between Ha’apai and Vava’u (student’s *t*-test, *p*>0.05). Of the corals sampled, host assemblage (i.e., freq.; Fig 1) differed significantly across archipelagos (S1 Table; *X*^2^ test, *p*<0.01), and this was mainly due to a relatively higher proportion of sampled colonies being the α *P*. *damicornis* genotype at Ha’apai (38.5% of the 52 genotyped colonies) compared to Vava’u (only 14% of the 56 genotyped colonies). Across the 115 colonies sampled, 108 were genotyped, and less than 1/3 of the sampled colonies were the model coral for research *P*. *damicornis*; the vast majority (72%) were instead *P*. *acuta* (Fig 1).

### Overview of the physiological data and PCA

**Fig 3:**
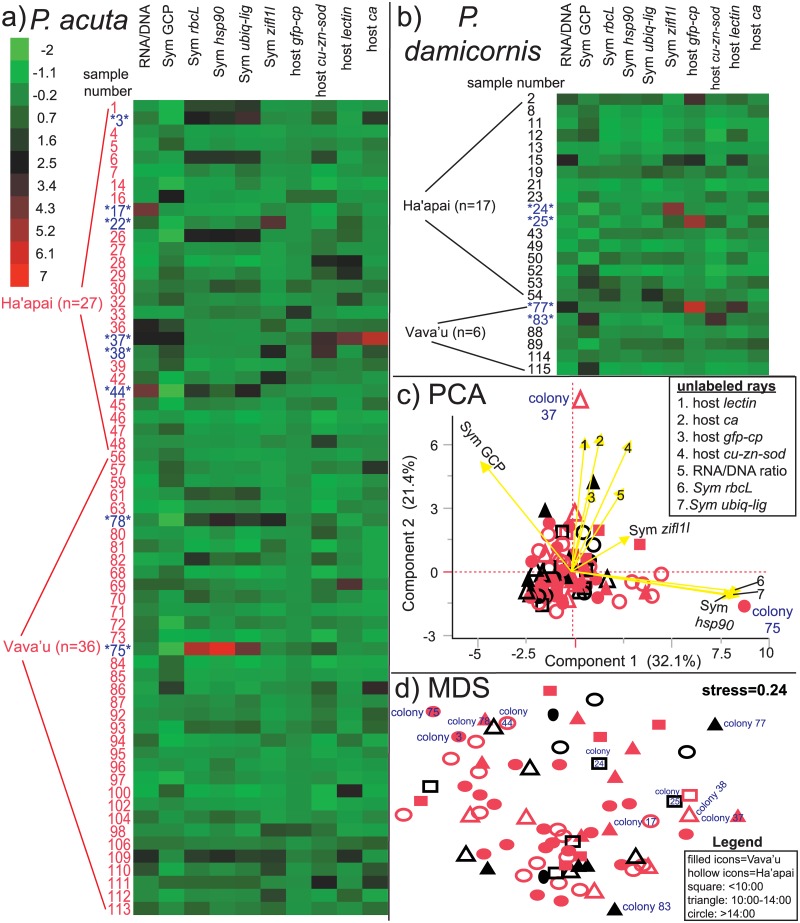
Heat maps and ordination analyses. Heat maps (as *z*-scores) were created for both the *Pocillopora acuta* (a; n = 63) and *P*. *damicornis* (b; n = 23) datasets. The 12 outliers identified (see Table 4.) are highlighted in blue. Principal components analysis (PCA) was performed to depict variation in the dataset (c), as well as to identify outliers, and the legend for this panel, which is shared with that of the multidimensional scaling (MDS) plot, is found in the bottom right corner of the figure. Two outliers have been labeled. For the loading scores, please see S1 Data. In the MDS plot (d), 11 of the 12 outliers have been labeled (colony 22 being the exception). The underlying Bray-Curtis similarity matrix can be found in S1 Data. For all panels, the species color code is as follows: colony number or icon in black = *P*. *damicornis* and colony number or icon in red = *P*. *acuta*.

Pocilloporid corals were sampled from 25 of the 59 surveyed reefs, and 88 and 108 were processed for all MSRV and genotyped, respectively. Heat maps were created to visualize data trends for the two dominant pocilloporid species sampled: *P*. *acuta* (Fig 3a) and *P*. *damicornis* (Fig 3b). For the actual data, rather than the *z*-scores depicted in such heat maps, please see S1 Data. Certain samples already stood out as being those demonstrating potentially aberrant behavior with this graphical approach alone; for instance, three red cells (*z*-score>5) can be seen for colony 75 (i.e., heat map score = 3; Fig 3a), potentially suggesting that this colony was behaving in a significantly different manner from other colonies with respect to the 10 MSRV. More rigorous, quantitative, and multivariate means of determining whether this colony, as well as others, were indeed outliers are discussed below.

**Table 4:** The 12 outliers identified in the Tonga dataset. Gene expression data have been presented as non-normalized (2^-Ct^*10^9^) in all but the last six rows; this allows for the back-calculation of the raw threshold cycle (Ct) values so that interested individuals can readily estimate the typical range of expression of each gene. Values representing aberrant levels for a particular parameter (*z*-score>2.5) have been highlighted in bold. When there was a statistically significant difference (student’s *t*-test, *p*<0.05) between the outlier and non-outlier averages for a parameter (instead using normalized gene expression data), the lower of the two values has been underlined. All samples hosted *Symbiodinium* of clade C only unless noted otherwise. The mean Mahalanobis distance did not differ between *Pocillopora damicornis* and *P*. *acuta* (student’s *t*-test, *p*>0.05). SA = surface area. GCP = genome copy proportion. Ma Dis = Mahalanobis distance. “.” = missing data.

Is-land/ sample	species	Coll-ection Time	Colony depth (m)	Color	Max. length (cm)	Planar SA (cm^2^)	Sym GCP	RNA/DNA	*Symbiodinium* genes (n = 4)				Host coral genes (n = 4)				MaDis	Heat map score
									*rbcL*	*zifl1l*	*hsp90*	*ubiq-lig*	*ca*	*lec-tin*	*cu-zn-sod*	*gfp-cp*		
**Ha’apai** (8/43 samples^a^ were outliers; 19%)																		
*TOHA01 (1/6 samples analyzed were outliers; 17%)*																		
3	*P*. *acuta*	14:45	25	normal	.	.	0.14^b^	1.0	**1170**	109	106.2	**35.0**	20130	25.4	329	122	4.2	2
*TOHA08 (1/5 samples analyzed were outliers; 20%)*																		
17	*P*. *acuta*	14:58	16	pale	33	458	0.26	**4.6**	208	370	2.54	5.45	3250	345	71.7	49.5	5.1	1
*TOHA09 (3/6 analyzed samples were outliers; 50%)*																		
22	*P*. *acuta*	8:55	19	pale	18	150	0.15	1.5	466	**1550**	26.0	14.2	6420	723	147	76.9	4.2	1
24	*P*. *damicornis*	9:06	10.5	normal	17	117	0.13	0.61	425	**1660**	11.8	13.3	1510	181	198	262	5.3	1
25	*P*. *damicornis*	9:12	5.8	normal	25	296	0.33	0.19	198	1550	70.1	11.3	21090	51.9	629	**13200**	5.7	1
*TOHA19 (3/5 analyzed samples were outliers; 60%)*																		
37	*P*. *acuta*	11:10	13	bleached	15	119	0.71	3.0	1150	548	88.3	27.2	**19200**	**523**	**280**	284	6.8	3
38	*P*. *acuta*	11:15	15	very pale	31	515	0.53	0.90	523	2044	51.9	19.2	7630	40.2	**378**	82.4	4.7	1
44	*P*. *acuta*	15:03	21.5	very pale	.	.	0.06^b^	**4.6**	574	80.5	35.9	**19.2**	7280	101.4	198	99.1	6.1	2
**Vava’u** (4/43 samples^a^ were outliers; 9%)																		
*TOVA40* (*1/6 samples analyzed were outliers; 17%)*																		
75	*P*. *acuta*	17:00	14.5	pale	38	520	0.08	0.83	**1150**	61.0	**150**	**22.1**	4380	88.3	374	2096	7.4	3
*TOVA35-round 2 (3/7 analyzed samples were outliers; 43%)*																		
77	*P*. *damicornis*	11:38	10	normal	15	112	0.33	2.8	488	644	38.4	13.6	2960	**690**	181	**3904**	6.2	2
78	*P*. *acuta*	11:43	11	pale	10	60	0.09	0.73	396	322	**76.9**	11.3	5650	122	268	228	4.8	1
83	*P*. *damicornis*	12:18	11	normal	11	64	**0.80**	0.50	415	76.9	36.7	9.83	1480	20.1	**147**	111	5.0	2
**Outlier avg. (normalized data; n = 12)**					21	241	0.30	1.8	10055	7056	1085	267	35000	1020	1140	4240	5.5	1.8
**Non-outlier avg. (normalized data; n = 74)**					16^d^	151^d^	0.33^c^	0.72^d^	4910^e^	1880	455^e^	135^e^	16300^e^	613^d^	564^e^	900^d^	2.4	0.1
***P*. *damicornis* outlier average (normalized data; n = 4)**					17	147	0.40	1.0	3360	8520	250	101	20280	1220	1230	11040	5.5	1.8
***P*. *damicornis* non-outlier average (normalized data; n = 19)**					14^d^	105^d^	0.36^c^	0.72^d^	4260^e^	2130^e^	367^e^	127^e^	14940^e^	558^d^	385^e^	1590^d^	2.4	0.1
***P*. *acuta* outlier average (normalized data; n = 8)**					24	304	0.25	2.1	13400	6330	1502	350	42300	921	1087	846	5.4	1.8
***P*. *acuta* non-outlier average (normalized data; n = 55)**					16^d^	167^d^	0.32^c^	0.72^d^	5140^e^	1800^e^	486^e^	137^e^	16800^e^^,^^f^	632^d^	626^e^	662^d^	2.4	0.1

^a^The total number of samples for which a Mahalanobis distance could be calculated.

^b^Possesses background levels of clade A *Symbiodinium*.

^c^root-transformed data.

^d^log-transformed data.

^e^rank-transformed data.

^f^*p* = 0.068.

PCA (Fig 3c) was used to depict the dataset in multiple dimensions in a manner that would also showcase the variability therein. However, it did not appear to be the best means of explaining variation between samples; the first two principal component (PC) axes accounted for less than 55% of the variation in the dataset, and most samples tended to cluster within the core, interior region of the plot. That being said, two colonies, 37 and 75, partitioned away from this central region. The latter showed separation along PC1, in which the *Symbiodinium hsp90*, *rbcL*, and *ubiq-lig* mRNAs possessed the highest positive loading scores (please see S1 Data for PCA loading scores and eigenvector composition.). In contrast to the *Symbiodinium* mRNA-dominated PC1, PC2 featured a number of host coral genes as those factors contributing the most positive loading scores (namely host *lectin* and *ca*), and colony 37 was separated from the core region of the plot along this PC. Both colonies 37 and 75 were found to be outliers based on their Mahalanobis distances and heat map scores (discussed below and in Table 4).

### MDS

As another means of visualizing similarity (or lack thereof) between the 86 analyzed samples, PRIMER was used to construct a Bray-Curtis similarity matrix (please see S1 Data.), and an MDS plot was created from this matrix (Fig 3d). ANOSIM was used to test the effects of the 14 environmental parameters on the resulting dataspace, and only an effect of time was documented (global *R* = 0.151, *p* = 0.001). However, such a temporal effect is not evident from the MDS plot (Fig 3d), and colonies of the three groupings of sampling times (<10:00, 10:00–14:00, and >14:00) appear intermixed. Furthermore, the stress was over 0.2, suggesting that these findings should be interpreted cautiously. Eleven of the twelve outliers discussed below (excluding colony 22, which is masked beneath the densest assemblage of icons in the plot) were labeled, and most appear around the periphery of the plot. Colonies 75, 77, and 83 were the three most different samples from each other (S1 Data), and all three of these were considered outliers (discussed below and in Table 4).

### Univariate statistical analyses and MANOVA

At the Bonferroni-adjusted univariate ANOVA α level of 0.004, few environmental factors were found to affect coral physiology (S1 Table). Max. colony length varied significantly across temperature and ALCC (S1 Table). Regarding temperature, colonies sampled between 26 and 27°C tended to be smaller than those sampled at lower temperatures (Tukey’s HSD, *p*<0.05 against 24–25°C and 25–26°C). Regarding the ALCC effect, corals sampled on reefs with ALCC of 20–30% (the low end of the spectrum) tended to be larger than those sampled from reefs of 40–50% and 50–60% ALCC (Tukey’s HSD, *p*<0.05 for each comparison). Polyp expansion varied significantly between species (*X*^2^ = 12, *p*<0.001), and *P*. *acuta* colonies were over 3-fold more likely than *P*. *damicornis* colonies to be characterized by expanded polyps during daylight hours (77 vs. 23%, respectively).

Only one MSRV, *Symbiodinium zifl1l* mRNA expression (pooled across both species; S1 Table), was significantly affected by any environmental parameter; specifically, its expression varied significantly between the three sampling time periods (1-way ANOVA, *p*<0.0001). Expression was highest in colonies sampled before 10:00, 2-fold lower in colonies sampled between 10:00 and 14:00 (Tukey’s HSD, *p*<0.05), and ~3.5-fold lower (relative to <10:00) in colonies sampled after 14:00 (Tukey’s HSD, *p*<0.05). The species-specific temporal expression patterns of this gene are discussed in more detail below.

**Fig 4:**
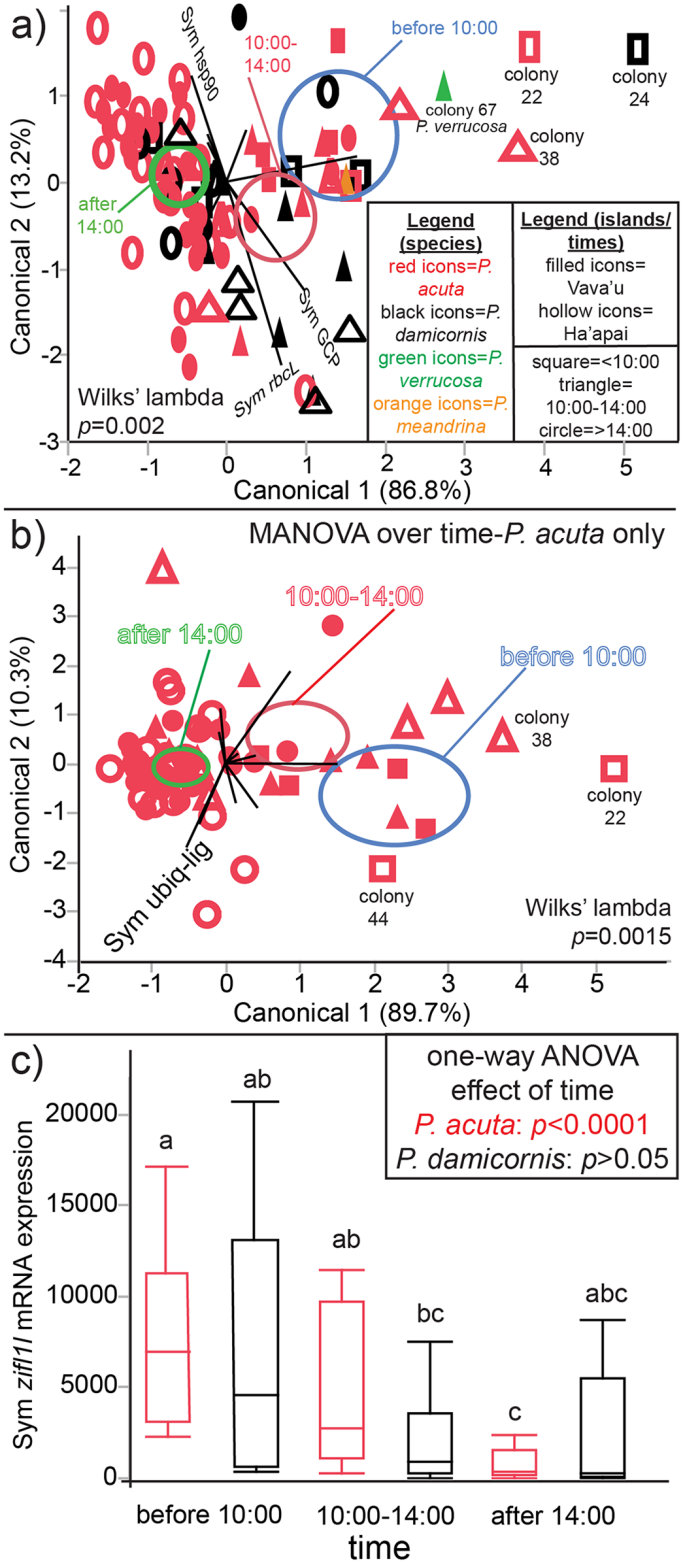
Temporal effects. Sampling time (three categorical groupings: <10:00, 10:00–14:00, and >14:00) was found to affect the multivariate molecular physiological response (a), though this was mainly due to temporal variation within the *Pocillopora acuta* dataset only (b). Several notable outliers have been labeled in the first two panels, and only a few biplot rays have been labeled in each due to spatial constraints. The blue, red, and green centroids represent 95% confidence for the <10:00, 10:00–14:00, and >14:00 sampling times, respectively. For the loading scores, please see S1 Data, and the legend for (a-b) is found in (a). The main driver of this multivariate temporal effect for *P*. *acuta* was the *Symbiodinium* zinc-induced facilitator-like 1-like (*zifl1l*) mRNA (see canonical scores in S1 Data.), and its expression has been plotted for each species at the three sampling time intervals (c). In this panel, the letters above the normal quantile plots reflect Tukey’s honestly significant difference groups (*p*<0.05) for the interaction of species (*P*. *acuta* vs. *P*. *damicornis*) and time (2-way ANOVA interaction effect, *p*<0.001). One-way ANOVA *p*-values (time effects) for each species analyzed in isolation have been provided in an inset.

When looking at the effects of the 14 environmental parameters on the multivariate response with MANOVA (S1 Table), only one environmental parameter, time, was found to significantly affect the centroid distribution. However, upon looking at the CCA plot of this result (Fig 4a), it is clear that the 95% confidence centroids do not encompass the majority of samples for any of the three categorical time groupings (<10:00, 10:00–14:00, and >14:00). In fact, three 10:00–14:00 samples (triangles) fall closer to the <10:00 centroid (blue). Colony 38 appears especially isolated along canonical axis (CA) 1. When performing individual MANOVAs for each of the two host species, *P*. *damicornis* and *P*. *acuta*, only the latter’s molecular signatures varied significantly over time (S2 Table); this is depicted graphically in Fig 4b. As when all data were considered, certain samples did not fit the trend; colony 38 again fell closer to the <10:00 sampling centroid despite the colony having been sampled at 11:15 (S1 Data). Colony 22 appears well separated from the other <10:00 samples along CA1, which was dominated by the *Symbiodinium zifl1l* mRNA (S1 Data; the biplot ray is unlabeled in Fig 4b due to spatial constraints.).

Given these results, the *zifl1l* mRNA expression data were plotted over sampling time for each species (Fig 4c), and Tukey’s *post-hoc* (HSD) differences were uncovered for *P*. *acuta* only; notably, expression of this gene peaked in the morning, gradually decreased at midday, and dropped dramatically at night. Additional univariate ANOVA results for *P*. *damicornis* and *P*. *acuta* analyzed separately can be found in the S2 Table. In general, findings paralleled those discussed above when both species were considered simultaneously in the data analysis (S1 Table). It is worth noting that the temperature and ALCC effects on the multivariate mean vector for *P*. *damicornis* (S2 Table) were due in both cases to the strong influence of two putative outliers: colonies 77 and 83 (discussed in more detail below).

### Outlier analysis

Two criteria were used to define outliers: 1) a Mahalanobis distance above the UCL of 4 and 2) a *z*-score above 2.5 for any MSRV (i.e., heat map score ≥ 1). It bears mentioning that this *z*-score criterion, and not a comparably low *z*-score threshold (i.e., <-2.5), was chosen because aberrantly low levels of any MSRV were rare; the lowest *z*-score in the entire dataset (-1.7) corresponded to a markedly low *Symbiodinium* density in one very pale *P*. *acuta* sample from Ha’apai. In contrast, maximum *z*-scores for each of the 10 MSRV ranged from 3 to 7; data were therefore highly skewed towards abnormally high values (Fig 3). Thirteen outliers were documented across the 86-sample subset analyzed for all 10 MSRV (Table 4); however, one (colony 67) represented the lone *P*. *verrucosa* sample in the dataset and, because it is unclear whether the qPCR assays are equally effective at amplifying gene homologs of this coral species, colony 67 was omitted from this list. The response variables that contributed most significantly to a sample being deemed an outlier are highlighted in bold in Table 4, and only temperature affected outlier frequency (S1 Table); however, this finding was driven by the fact that three outliers were sampled from reefs for which no temperature data existed; therefore, the “no data” category was over-represented in the contingency table (results not presented herein; see S1 Data.). For this reason, this temperature effect is not discussed further.

When calculating Mahalanobis distances for each species individually, one additional *P*. *acuta* colony was found to be an outlier: colony 16 had an aberrantly high *Symbiodinium* density; however, as this sample possessed a mixed assemblage of *Symbiodinium* clades (A+C), it is possible that differential primer binding to gene homologs of the two clades biased the nucleic acid quantification; therefore, this sample was excluded from the outlier analysis. One additional *P*. *damicornis* outlier was also uncovered when considering each species in isolation; colony 15 demonstrated an aberrantly high RNA/DNA ratio. However, as its distance of 4.0 was only slightly higher than the *P*. *damicornis*-only UCL of 3.8, and its Mahalanobis distance was below the UCL when all data were analyzed together, this sample was also excluded from the total outlier count.

### Outliers vs. non-outliers

**Fig 5:**
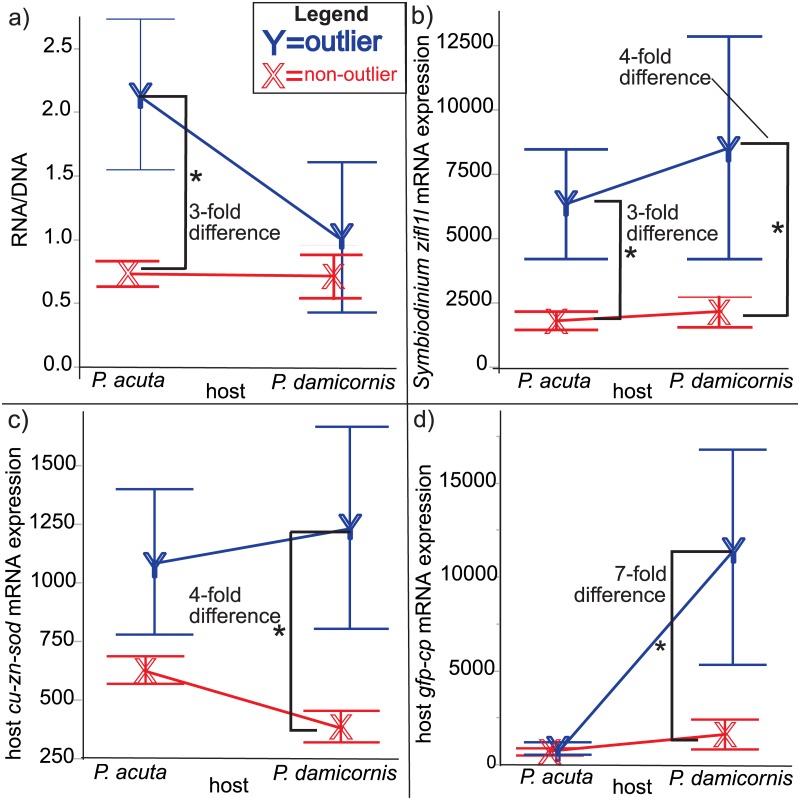
Species-specific differences between outliers and non-outliers. Average levels of the four molecular-scale response variables that differed significantly between outliers and non-outliers were plotted separately for each species and included the RNA/DNA ratio (a), the *Symbiodinium* zinc-induced facilitator-like 1-like (*zifl1l*) mRNA (b), the host coral copper-zinc superoxide dismutase (*cu-zn-sod*) mRNA (c), and the host coral green fluorescent protein-like chromoprotein (*gfp-cp*) mRNA (d). Error bars represent standard deviation. Asterisks (*) denote within-species differences (Tukey’s honestly significant difference, *p*<0.05). One host coral gene whose expression level differed significantly between outliers and non-outliers when data were pooled across species, carbonic anhydrase (*ca*; see Table 4.), was not found to differ significantly across outlier status within species and so has not been included in the figure.

To understand the overall differences between the 12 outliers and the 74 non-outliers, a series of student’s *t*-tests were first performed; several notable differences were unveiled (Table 4). First, the outliers had greater than 2-fold higher RNA/DNA ratios (Table 4). Furthermore, outliers demonstrated higher expression levels of four of the eight target genes, including *Symbiodinium zifl1l* (~4-fold difference) and host coral *ca* (~2-fold difference), *cu-zn-sod* (~2-fold difference), and *gfp-cp* (~5-fold difference). However, when looking at differences between outliers and non-outliers individually for each of the two species sampled, *P*. *acuta* and *P*. *damicornis* (Table 4 and Fig 5), it is clear that the RNA/DNA ratio difference mentioned above is mainly attributed to the former species (Fig 5a); this ratio did not differ significantly between outlier and non-outlier *P*. *damicornis* colonies. In contrast, the host coral gene expression differences between outliers and non-outliers were mainly driven by *P*. *damicornis*; the expression of no host coral gene differed between outliers and non-outliers for *P*. *acuta* (Fig 5c and 5d). Only one gene was expressed at significantly different levels between outliers and non-outliers for both host coral species; *Symbiodinium zifl11* was expressed at 3- and 4-fold higher levels in outliers of *P*. *acuta* and *P*. *damicornis*, respectively, relative to non-outliers (Fig 5b).

### Predictor screening and the variability index

**Fig 6:**
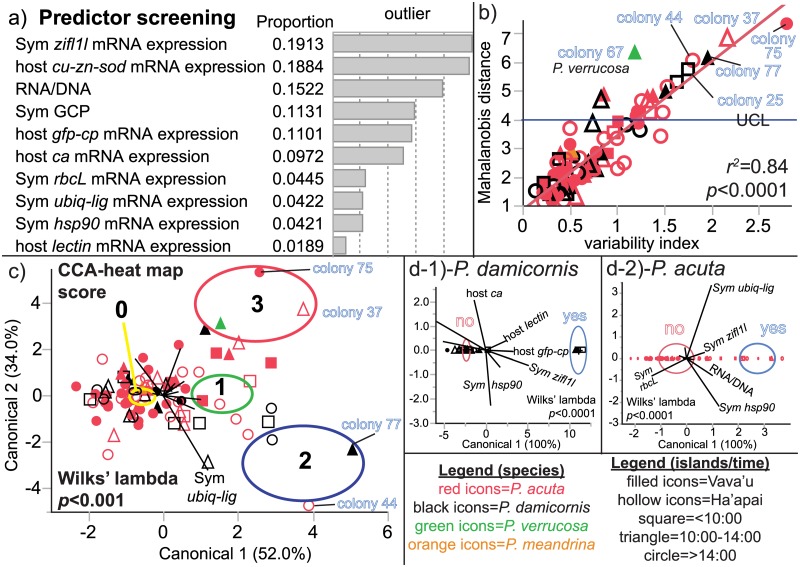
Multivariate analysis of the 12 outliers. JMP’s (ver. 12.0.1) “predictor screening” function was used to rank the molecular-scale response variables in terms of their contribution to the proportional variation between outliers (n = 12) and non-outliers (n = 74; a). There was a statistically significant correlation between the Mahalanobis distance and the variability index (b), and the legend for this panel, which is shared with (c-d), is found at the bottom right corner of the figure. Several notable outliers have been labeled. Canonical correlation analysis (CCA) was used to model the differences between samples characterized by heat map scores of 0, 1, 2, or 3 (c). Those of the latter two groups were typically considered outliers (see Table 4.), of which several have been labeled. The yellow, green, blue, and red centroids in (c) represent 95% confidence for the heat map score groups of 0, 1, 2, and 3, respectively. In (d), CCAs testing the differences between outliers (“yes;” blue 95% confidence centroids) and non-outliers (“no;” red 95% confidence centroids) were performed individually for each species sampled: *Pocillopora damicornis* (d-1) and *P*. *acuta* (d-2). Not all biplot axes have been labeled in (c-d), and icons have been presented in reduced size due to the extensive degree of overlap amongst samples within each group.

As another means of uncovering the response variables that best differentiated outliers from non-outliers, JMP’s predictor screening function was used to rank the response variables in terms of their proportional contribution to the cumulative difference between outliers and non-outliers (Fig 6a). *Symbiodinium zifl1l* mRNA expression ranked highest, accounting for nearly 20% of the cumulative difference between outliers and non-outliers. This is unsurprising given the statistically significant, ~3.5-fold difference between outlier *zifl1l* expression and non-outlier expression of this gene mentioned above (Table 4 and Fig 5b). Similarly, the parameters for which there was no statistically significant difference in the outlier vs. non-outlier student’s *t*-tests of Table 4 ranked lowest: host *lectin* (~2% of the cumulative difference) and *Symbiodinium hsp90* (4%) and *ubiq-lig* (4%).

There was a positive, linear, and statistically significant correlation (*r*^2^ = 0.84, *p*<0.001) between the Mahalanobis distance and the variability index (the standard deviation calculated across *z*-scores for all 10 MSRV for each sample; Fig 6b). In other words, those samples whose multivariate means calculated across the response variables assessed deviated most from the global mean centroid (i.e., high Mahalanobis distance) were also those demonstrating the greatest variation between the response variables (i.e., high variability index). The outliers had, on average, nearly 3-fold higher variability amongst the response variables (1.72±0.42) than the non-outliers (0.60±0.31); this difference was statistically significant (student’s *t*-test *p*<0.0001). The variability index did not vary across any environmental parameter (results not presented herein; see S1 Data.).

### Outlier CCA

MANOVA+CCA featuring the 10 MSRV was also used to determine which parameters (or combinations thereof) best separated samples with high heat map scores (i.e., the outliers) from non-outliers (Fig 6c), and there was good separation along CA1 (52%). The *Symbiodinium zifl1l* mRNA was the most positively weighted parameter along CA1, whereas the negative relationship between *Symbiodinium rbcL* and *ubiq-lig* mRNA expression was most important for separation along CA2 (please see S1 Data for canonical scores.). Four outliers were well separated along CAs 1 and 2 (labeled in Fig 6c). Samples with heat map scores of 2 and 3 (all of which were outliers) were separated from samples with heat map scores of 0 along CA1, whereas the former two groups were separated from each other along CA2 (34%). When looking at multivariate differences between outliers and non-outliers for the two species analyzed in isolation, *Symbiodinium zifl1l* was strongly positively weighted for both *P*. *damicornis* (Fig 6d-1) and *P*. *acuta* (Fig 6d-2). However, for the former species, host *gfp-cp* mRNA expression was more important in the separation of outliers from non-outliers (S1 Data). This is due to the fact that expression of this gene was 7-fold different between outliers and non-outliers for *P*. *damicornis*, whereas it did not differ in expression between outliers and non-outliers for *P*. *acuta* (Fig 5d).

## Discussion

In addition to serving as the first molecule- to ecosystem-scale dataset for Tonga’s reef corals and coral reefs, the dataset featured herein was characterized by several interesting properties. First, the majority of the data were “negative;” there were, in general, few coral physiological differences across the environmental gradients assessed. Nevertheless, a suite of MSA were able to uncover outlier coral colonies within the dataset, and the cellular properties diagnostic of statistically aberrant behavior differed across the two main pocilloporid species sampled, *P*. *damicornis* and *P*. *acuta*. Outlier characteristics also differed from those of aberrantly behaving pocilloporid coral colonies of Fiji. The molecular physiology of *P*. *acuta* varied more over time than did *P*. *damicornis*, and this was due mainly to temporally dynamic *Symbiodinium* gene expression fluctuations in the former species (but not the latter). This gene mRNA-level difference between these closely related coral species may be related to mode of energy acquisition and have implications for the relative ability of each species to acclimatize to future changes in their environments. All points mentioned in this introductory paragraph are discussed in greater detail below.

As mentioned above, a striking feature of this dataset is the large quantity of negative data. Only 1 of the 10 MSRV, the *Symbiodinium zifl1l* mRNA, was significantly affected by any environmental parameter upon having Bonferroni-adjusted the ANOVA α levels. This *zifl1l* finding is discussed in more detail below. We surmise that the preponderance of null findings was largely driven by high variation between samples. The coefficients of variation (located under “sample info” in S1 Data) for the 10 MSRV ranged from ~50 to ~250% and averaged 130%; the standard deviation was higher than the mean for all but three response variables. Although 86 coral colonies were analyzed in detail, representing a much larger sample size than other coral molecular ecophysiology surveys (e.g., 25 colonies [9]), the number of colonies within any one statistical “bin” was relatively small in certain cases. For instance, only one and two colonies were collected shallower than 5 m and deeper than 30 m, respectively; these low sample sizes, and the associated high variation across colonies, made it unlikely that depth effects would be documented in particular. Similarly, only five colonies were sampled from barrier reefs, versus 29 and 52 from fringing and patch reefs, respectively.

Were each of the two target species found at similar abundances at all depths, archipelagos, reef types, reef zones, etc., a more balanced sampling design would have been achieved; such would certainly have increased our statistical power. Nevertheless, as we used the protocol outlined herein to survey reefs and sample pocilloporid corals from a number of nations across the Indo-Pacific as part of the GRE [31–32], we might eventually obtain a large enough dataset, with sufficient sample sizes within each statistical bin (i.e., depth, reef type, etc.), to uncover statistically significant, biologically meaningful differences in certain MSRV across the ~10–15 environmental characteristics routinely documented. As mentioned in more detail below, though, certain environmental parameters, such as nutrient levels, were not measured during the GRE, and such seawater properties may instead be important in driving variation in coral cell physiology.

Despite the fact that few MSRV were affected by the environmental properties assessed, a number of outliers were nevertheless detected across the 86 *P*. *damicornis*+*P*. *acuta* samples analyzed for the 10 MSRV; 12 (14%) were found to be outliers based on an aberrancy detection system featuring both univariate (i.e., *z*-score [analyzed individually for each parameter] > 2.5) and multivariate (i.e., Mahalanobis distance > 4) statistical approaches. A variety of MSA validated these findings. For instance, 2 of the 12 outliers were also identified by PCA, and the 4 most deviant points in the MDS plot were all flagged as outliers. A MANOVA of the effects of time also led to the separation of certain outliers from the normally behaving colonies (e.g., colony 38). This corroboration between techniques in identifying samples that were exhibiting statistically aberrant behavior at the time of sampling instills greater confidence in the assignment of these samples as outliers and confirms our hypothesis put forth previously [31–32] that multiple MSA can be used to collectively determine which corals are statistically abnormal, even when the underlying response variables analyzed in isolation do not vary across environments.

Interestingly, outlier frequency was not influenced by environment; outliers were, for instance, equally likely to be found on reefs with low coral cover as those with high coral cover. This contrasts with what had been hypothesized and suggests that there is not a clear relationship between certain ecosystem-scale measures of reef condition (e.g., algal abundance) and organismal-scale indices of reef coral performance; this was also the case in the Austral Islands of French Polynesia [32], the Cook Islands [32], and Fiji [31]. It is worth noting that for corals of Fiji, light (which was not measured herein) had no affect on gene expression or outlier frequency. Although it is tempting to postulate, then, that outlier distribution is decidedly random, perhaps biotic or abiotic parameters that were not measured herein or in these prior works, such as coral microbiomes or seawater nutrient levels, respectively, are instead more important in eliciting aberrant coral behavior. Therefore, more thorough seawater quality and coral biopsy evaluations may be warranted in future works to conclusively determine the factors that cause certain corals to behave in a statistically different manner from nearby conspecifics or congenerics of presumably similar environmental history, size, and age.

Regarding the properties of the outliers uncovered herein, they were characterized by 2-fold higher RNA/DNA ratios, as well as higher mRNA expression levels of several genes previously found to be implicated in the coral stress/acclimation response: *Symbiodinium zifl1l* and host coral *ca*, *cu-zn-sod*, and *gfp-cp*. However, only the former *Symbiodinium* gene, which is involved in zinc transport [38], differed significantly between outliers and non-outliers for both target species: *P*. *acuta* and *P*. *damicornis*. Zinc concentrations were not measured in seawater or coral tissues, though the aberrant behavior associated with the 12 outliers could have manifested in, or been driven by, metabolic dysfunction within the coral holobiont (*sensu* [2]). Zinc is necessary for a number of cellular processes, such as gene expression itself, and if the hosts’ ability to uptake zinc from the surrounding seawater or via heterotrophic feeding was compromised, then this could have resulted in an increase in the number of zinc transporters at the host-*Symbiodinium* interface in the case of the 12 outliers. The potential influence of host coral heterotrophy on *Symbiodinium zifl1l* mRNA expression is discussed in more detail below.

Differences in light quality/quantity could also have accounted for variation in *zifl1l* expression, especially given the dramatic drop in expression over the course of the day in *Symbiodinium* populations within *P*. *acuta*, a temporal trend identical to that of *Symbiodinium* within pocilloporid corals of Fiji’s Lau Archipelago [31]. This temporal variation in zinc transporter expression, which was not observed in *Symbiodinium* populations housed within Tongan *P*. *damicornis* colonies, may indeed attest to the complex, dynamic, dual-compartmental metabolism of coral-dinoflagellate endosymbioses [2]. The fact that *Symbiodinium zifl1l* mRNA expression, and molecular physiology in general, varied significantly over time for *P*. *acuta*, but not *P*. *damicornis*, is worth addressing in greater detail. *P*. *acuta* and *P*. *damicornis* are closely related [26], appear similar *in situ* (see images on coralreefdiagnostics.com, Dryad, and Fig 2.), and were characterized by similar outlier frequency herein (13 and 17% of all colonies were outliers, respectively). However, they are typically found in different habitats [5], and they demonstrated different behavioral patterns herein; *P*. *acuta* was over 3-fold more likely to have its tentacles expanded during the daylight hours than *P*. *damicornis*. This may suggest that *P*. *acuta* is more adept at, or relies more heavily on, heterotrophy than does *P*. *damicornis*; such a feeding strategy difference may have resulted in differential zinc uptake between the two species, thereby accounting for, in part, the varying fluctuation patterns of *Symbiodinium zifl1l* expression.

Future works should, then, attempt to observe relative differences in autotrophy vs. heterotrophy in these two species, as such will have significant implications for their ability to recover from bleaching. For instance, species that are more adept at switching from autotrophy to heterotrophy have been shown to fair better in response to climate change scenarios than those less adapted to making such a metabolic transition [39]. Furthermore, since there is little congruency between mRNA and protein expression in *Symbiodinium* [40–41], it will be necessary to instead profile ZIFL1l *protein* concentration and activity over diel cycles in such future studies in order to conclusively determine the role of this molecule in zinc homeostasis and metabolism in the pocilloporid coral-*Symbiodinium* mutualism.

A similar outlier detection scheme was used to identify corals displaying aberrant behavior in Fiji’s Lau Archipelago [31]. In that work, however, a different suite of response variables best modeled differences between corals behaving normally and those deemed as outliers (11 of the 70 Fijian colonies analyzed in detail [16%] were outliers, a statistically similar percentage to that of the Tonga dataset [14%].). In contrast to the Tonga dataset, though, the Fijian outliers possessed lower densities of *Symbiodinium* and higher expression levels of several genes encoding proteins involved directly in the cellular stress response, notably *ubiq-lig*. Therefore, it is more likely that the outliers detected in the Fiji dataset were indeed experiencing stress, whereas it is unclear whether the 12 Tonga outliers were stressed at the time of sampling. For one, neither *Symbiodinium* density nor *Symbiodinium ubiq-lig* mRNA expression varied significantly between outliers and non-outliers in the Tonga colonies; instead, as mentioned above, *Symbiodinium zifl1l* and two host coral genes, *cu-zn-sod* and *gfp-cp*, were more important in separating statistically normally behaving corals from those demonstrating aberrant molecular physiology. Amongst these three genes, only *cu-zn-sod* encodes a protein involved in the cellular stress response as it is classically understood [42], and one completely bleached colony (#37) expressed aberrantly high levels of this gene; given the well documented relationship between reactive oxygen species (ROS) levels and coral bleaching [43], elevated expression of a gene encoding a protein involved in ROS scavenging (i.e., CU-ZN-SOD) by a coral undergoing bleaching is unsurprising.

Although reef corals experiencing stress-inducing temperatures tend to down-regulate expression of *gfp-cp* [29], which encodes a protein involved in light absorption [44], those exposed to high light levels do the opposite [24]. Therefore, abnormally high or low levels of expression of this gene could be indicative of aberrant behavior stemming from unfavorable environmental conditions. Only two of the four *P*. *damicornis* outliers, though (colonies 25 and 77), actually expressed aberrantly high levels of the *gfp-cp* mRNA. The former colony was located at only 5.8 m, and so the high irradiance associated with such a shallow depth could have explained the high *gfp-cp* mRNA expression in this colony. However, as neither irradiance nor photosynthetically active radiation was measured at the exact site of coral collection, the role of light in driving the abnormally high expression of *gfp-cp* in this sample cannot be elucidated. Indeed, an understanding of the effects of abnormally high irradiance on coral molecular biomarker profiling represents a fruitful line of investigation for future study.

Given the prohibitive expense (~$US600,000) of returning to the same reefs during a period of environmental disturbance (e.g., an unusually high temperature spike), it is currently not possible to determine whether the aberrant sub-cellular behavior of the 12 outliers is diagnostic of stress, or, in contrast, highlights a degree of phenotypic plasticity that might actually be associated with an enhanced degree of resilience. If outliers are ultimately less resilient than non-outliers to changes in their environment, then this suite of MSRV, and particularly the stress-targeted genes, may have diagnostic potential (though see additional caveats raised in the Introduction.). It deserves mention that in humans, a high gene expression variability index, which was documented herein for the 12 outliers, is a hallmark of certain cancers and numerous other diseases [45]. If, on the other hand, outliers are demonstrated to be *more* resilient than normally behaving corals, then it is possible that the “front-loading hypothesis” first uncovered by Mayfield et al. [27] and later corroborated by Barshis et al. [46] is a better explanation for the composite findings of the dataset; high expression levels of stress-targeted genes, in particular, may be a strategy for countering future environmental changes. In this case, the MSRV assays+MSA approach outlined herein could instead be used to identify particularly robust coral colonies; such might be the best candidates for reef restoration projects [47].

Finally, if outliers and non-outliers respond similarly to future environmental changes, then the dataset may simply attest to a broad degree of phenotypic plasticity within colonies of both target pocilloporid species; such a trait may be advantageous given the currently unstable abiotic milieu in which these coral reside [48]. In conclusion, although it is currently premature to forecast which of the colonies sampled herein will be most likely to bleach as sea temperatures rise, we nevertheless believe that these baseline ecological-, physiological-, and molecular-scale data for coral reefs and scleractinian corals of Tonga will be useful to researchers working in the South Pacific, and particularly scientists wishing to monitor and track the health of Tongan corals reefs in this era of changing ocean chemistry. When re-surveying these reefs in the future, and particularly during periods of environmental stress, researchers should attempt to not only uncover whether the outlier assignment exercise presented herein is able to identify corals that are prone to, for instance, bleaching, but they can also directly test the aforementioned hypothesis that *P*. *acuta* fairs better during bleaching events due to its hypothetically elevated capacity for heterotrophy.

## Supporting information

S1 File Supporting information. This document contains a brief introduction to the Tonga mission, which was part of the Khaled bin Sultan Living Oceans Foundation’s “Global Reef Expedition.” In addition to supplementary methods on groundtruthing and coral reef surveys, this document also contains supplemental results, including site descriptions for nearly all sites surveyed.
(DOCX)

S1 Data Online supplemental data file. This spreadsheet contains all data presented in the manuscript, including environmental data (worksheet 1) and molecular-physiological data from the sampled corals (worksheet 2). It also includes the results of the multivariate statistical analyses (MSA), including principal components analysis (PCA) and multivariate ANOVA (MANOVA; worksheet 3). Finally, a Bray-Curtis similarity matrix has also been included (worksheet 4).
(XLSX)

S1 Table Univariate statistical analysis of the Tonga dataset-I: Host species analyzed together. The values below the environmental parameters (EP; top row) represent the number of categorical groupings. *X*^2^ tests and 1-way ANOVAs were used to analyze the frequency (freq.) and molecular+physiological data, respectively. Comparisons that were statistically significant at the Bonferroni-adjusted α levels of 0.013, 0.013, 0.013, 0.004, and 0.013 for the host freq. *X*^2^ tests, outlier freq. *X*^2^ tests, polyp expansion freq. *X*^2^ tests, molecular-scale response variable (MSRV) ANOVAs, and multivariate ANOVAs (MANOVA; Wilks’ lambda was calculated.), respectively, are highlighted in green, and marginally significant *p*-values have been highlighted in yellow. The *P*. *verrucosa* (n = 1) and *P*. *meandrina* (n = 1) samples were excluded from the analysis. ALCC = average live coral cover. Sym = *Symbiodinium*. NS = not statistically significant. NA = not applicable.
(DOCX)

S2 Table Univariate statistical analysis of the Tonga dataset-II: host species analyzed separately. The values below the environmental parameters (EP; top row) represent the number of categorical groupings. *X*^2^ tests and 1-way ANOVAs were used to analyze the frequency (freq.) and molecular+physiological data, respectively. Comparisons that were statistically significant at the Bonferroni-adjusted α levels of 0.013, 0.013, 0.004, and 0.013 for the outlier freq. *X*^2^ tests, polyp expansion (expan.) freq. *X*^2^ tests, molecular physiological response variable (MPRV) ANOVAs, and multivariate ANOVAs (MANOVA; Wilks’ lambda was calculated.), respectively, are highlighted in green, and marginally significant *p*-values have been highlighted in yellow. ALCC = average live coral cover. Sym = *Symbiodinium*. NS = not statistically significant.
(DOCX)
