# Supplementary material for: Biomarker profiling in reef corals of Tonga’s Ha’apai and Vava’u archipelagos
Source: PLoS One. 2017 Nov 1;12(11):e0185857. doi: 10.1371/journal.pone.0185857 (PMC5665425; doi:10.1371/journal.pone.0185857)
Supplement: S1 File — This document contains a brief introduction to the Tonga mission, which was part of the Khaled bin Sultan Living Oceans Foundation’s “Global Reef Expedition.” In addition to supplementary methods on groundtruthing and coral reef surveys, this document also contains supplemental results, including site descriptions for nearly all sites surveyed. (DOCX) [file pone.0185857.s001.docx]

**Mayfield et al. supporting information-table of contents**

**Summary of Tonga mission: Page 2**

**Supplemental methods: Pages 3-5**

**Groundtruthing: Pages 3-4**

**Coral reef surveys: Pages 4-5**

**Supplemental results: Pages 6-30**

**Average live coral cover: Page 6**

**Site descriptions: Pages 6-28**

**Ha’apai: Pages 6-19**

**Vava’u: Pages: 19-26**

**Niuatoputapu: Pages: 26-28**

**Summary of Tonga mission**

Between September 10 and October 3, 2013, the Khaled bin Sultan Living Oceans Foundation (KSLOF) conducted a research mission to the Kingdom of Tonga, focusing on coral reefs surrounding the islands in the Ha'apai (Sept. 11-21), Vava’u (Sept. 22-28), and Niuatoputapu (Sept. 29-Oct 1) island groups. The mission included coral reef surveys, scleractinian coral health assessments (described in the main manuscript text), and habitat mapping. The mission involved not only local Tongan scientists, but also researchers from Nova Southeastern University (USA), University of the Azores (Portugal), University of the Philippines, NOAA/University of Miami (USA), the Atlantic and Gulf Rapid Reef Assessment Program (AGRRA; USA), and the National Museum of Marine Biology and Aquarium (NMMBA; Taiwan). The objectives of the mission were to:

1. Identify and characterize shallow marine habitats and develop habitat and bathymetric maps.
2. Evaluate the composition, structure, and health of coral reefs using a standardized assessment protocol.
3. Evaluate the effects of environmental stressors on coral health by profiling sub-cellular biomarker expression patterns (see main manuscript text for details.).
4. Assess the diversity, abundance, and population structure of fishes, corals, and other invertebrates and algae, including commercially valuable species.
5. Document the impacts of broad-scale disturbances and patterns of recovery, with an emphasis on tsunami damage in Niuatoputapu.
6. Measure ocean pH and determine its effects on coral growth (using cores taken from massive coral species).
7. Describe the types of dinoflagellate endosymbionts in reef-building corals and characterize their photosynthetic efficiency under different environmental conditions.

Topics highlighted in yellow shading are addressed in this manuscript. The remaining objectives and their associated datasets will be featured in future works.

**Supplemental methods**

**Groundtruthing**

**Summary***.* A total of 2,212 km^2^ of WorldView-2 satellite imagery was acquired. To characterize shallow marine habitats, 524 videos (“drop cameras”) and 1.6 million soundings were taken across the three locations (covering a distance of 357 km). Geo-referenced WorldView-2 imagery was used to 1) plan bathymetric tracks for sampling and 2) locate habitats of interest for dropping video cameras. The bathymetric information will be used to calibrate a model that will assign a depth value to each pixel in the imagery data. Similarly, information from the camera drops will be used to assign a habitat type to the imagery data. Together, detailed maps of bathymetry and habitat type will be produced for the three locations visited and should be freely available in 2018 on the following website: <http://maps.lof.org/lof>.

**Habitat mapping and groundtruthing**. Using multispectral satellite imagery obtained from the DigitalGlobe WorldView-2 satellite, high resolution bathymetric maps and habitat maps are being created for shallow coral communities. Groundtruthing efforts necessary to develop these maps required 1) aerial surveys of each island’s coastline and adjacent shallow marine habitats, 2) continuous bathymetry measurements, 3) drop video camera analysis, 4) characterization of sediment and hard substrates and habitat features using two acoustic, sub-bottom profilers (Stratabox and Hydrobox), and 5) fine-scale photo-transect surveys. Each approach is described in more detail below.

**Satellite imagery***.* As mentioned above, 2,212 km^2^ of WorldView-2 (8-band) satellite imagery were acquired for this project. The satellite images had a spatial resolution of 2 m by 2 m (i.e., each pixel covered a 4 m^2^ area.), enabling real-time navigation in the field to locate features of interest and to avoid dangerous features such as emergent reefs. In order to navigate, the team used these scenes in conjunction with a differential GPS device (dGPS). The imagery is being used in conjunction with groundtruthing data to create bathymetric and benthic habitat maps.

**Benthic videos***.* An underwater video camera attached to a cable (called a “drop cam”) was used to acquire videos of the benthic communities at each survey site. At each point, the drop cam was held from the survey boat, enabling it to glide along the sea floor as it recorded video for 15 to 60 s. During this time, the laptop operator watched the video in real-time and directed the drop cam operator to raise or lower the camera in order to prevent damage to marine life. The video was recorded on a ruggedized laptop, and the geographic position, time, date, boat heading, and boat speed were burned into the video file. Drop cam deployment was limited to depths above 40 m due to the length of the cable (50 m). The videos acquired are being used to create benthic habitat maps by providing information needed for the development of habitat classification schemes and training of classification models. At least 30 drop cam videos were gathered per day.

**Acoustic depth soundings***.* Depth soundings were gathered along transects between survey sites using Hydrobox, a single-beam acoustic transducer developed by SyQwest. The instrument emitted three pings per second. Depths were estimated based on the time the return required for the return pulse to reach the sounder’s head. Geopositional data were simultaneously acquired by the dGPS unit. The estimated depth values and their geographic location were recorded in the ruggedized laptop. The soundings were used to develop a water-depth derivation model, which is based on the spectral attenuation of light in the water column. The final topographic map will have the same spatial resolution as the satellite imagery. An average of 100,000 acoustic depth soundings was gathered during a full work day.

**Acoustic sub-bottom profiling**. Profiles of the seafloor’s sub-bottom were also gathered along transects using the Stratabox acoustic sounder (developed by SyQwest). Similar to the bathymetric soundings, the sub-bottom profile emits an acoustic ping that reflects off the seafloor. However, the pulse has a lower frequency (3.5 Khz), enabling it to penetrate the seafloor. The instrument provides observations on stratal geometry beneath the seafloor along the transect lines, allowing estimates of Holocene reef-growth and sediment accumulation to be made. Geopositional data for each ping were simultaneously acquired by a dGPS unit. Profiles were run shore-perpendicular to capture the geometry of the bank flanks and spanned a depth range of 300 m to 5 m. Total transect length varied with the slope’s angle; steeper slopes resulted in shorter transect lines.

**Coral reef surveys**

**Summary***.* A total of 203 fish transects, 365 benthic surveys, 311 coral assessments, and 355 photo-transects were completed across the 59 reef sites, and surveys were typically performed from 5 to 30 m depth. The population structure and health of 27,308 corals (4 cm or larger) were also assessed. Roving surveys for commercially important macroinvertebrates were also undertaken. The details of all surveys methods are described below.

**Fish assessments**. Abundance and size structure data were collected for over 200 species of fish, and species that have major functional roles on reefs or are major fisheries targets were prioritized. Reef fish were assessed along 4 m x 30-m belt transects. A T-square marked in 5-cm increments was used to gauge fish size. At least six transects were conducted by each fish survey diver (typically two) per site. A roving survey was also completed to assess the total diversity and relative abundance (rare, common, vs. abundant) of reef fish at each site. These data will be presented in a future work.

**Benthic cover***.* Cover of major functional groups (corals identified to genus, sponges, other invertebrates, and six groups of algae including 1) macroalgae, 2) crustose coralline algae [CCA], 3) erect coralline algae, 4) fine turfs, 5) turf algae with sediment, and 6) cyanobacteria) and substrate type (hardground, sand, mud, rubble, recently dead coral, bleached coral, and live coral) were assessed along 10-m transects using either recorded observations or photographic assessments. Recorded observations involve a point-intercept method whereby the organism and substrate were identified every 10 cm along a 10-m transect (100 points/transect), with a minimum of six transects examined per location. When possible surveys were carried out at 30, 25, 20, 15, 10, and 5 m.

**Photographic assessments***.* A 10-m long transect tape was extended along depth contours at 30, 20, 15, 10, and 5 m. Continuous, digital, still photographs were taken of the reef substrate from a height of approximately 0.6-0.75 m above the substrate, using a 1-m bar divided into 5-cm increments placed perpendicular to the transect tape as a scale bar. Approximately 20 photographs were taken per transect to allow for overlap between adjacent images, and two photo transects (each 10 m in length) were conducted per depth. Images were downloaded onto a computer, and benthic community composition, coral cover and size (as planar surface area [SA]) cover of other organisms, and substrate type were analyzed using Coral Point Count (CPCE) software developed by the National Coral Reef Institute (NCRI). Cover was determined by recording the benthic attribute located directly below random points (30-50 points per photograph). Planar SA was measured by tracing the outline of individual corals. A total of 199, 120, and 36 photo-transects were carried out at Ha’apai, Vava’u, Niuatoputapu, respectively.

**Coral surveys***.* A combination of quantitative methods, including belt transects, point- intercept transects, radial plots, and quadrats were used to assess corals, other benthic organisms, and fish. Five measures were recorded for corals: 1) benthic cover (point intercept; see above.), 2) diversity and abundance (by genus, except for certain common species), 3) size class distributions, 4) recruitment, and 5) condition. Additional information was collected on causes of recent mortality, including signs of coral disease and predation. Assessment of corals smaller than 4 cm was done using a minimum of five 0.25 m^2^ quadrats per transect, with each quadrat located at fixed, predetermined intervals (2, 4, 6, 8, and 10 m; alternating between the right and left sides of the transect). Recruits were identified in both point-intercept surveys and belt transects and divided into two categories: 1) corals up to 2 cm in diameter and 2) those 2-3.9 cm in diameter.

Coral population structure and condition were assessed within belt transects (each 10 x 1 m), with a minimum of two transects performed per depth. Each coral 4 cm or larger was identified (to genus at minimum), and its growth form was recorded. Visual estimates of tissue loss were recorded for each colony over 4 cm in diameter using a 1-m bar marked in 1-cm increments (for scale). If the coral exhibited tissue loss, estimates of the amount of remaining tissue, percent that recently died, and percent that died long ago were made based on the entire colony surface area. Tissue loss was categorized as “recent mortality” (occurring within the last 1-5 days), “transitional mortality” (filamentous green algae and diatom colonization; 6-30 days), or old mortality (>30 days).

For each coral with partial or whole colony mortality, the cause of mortality was identified if possible. The diagnosis included an assessment of the type of disease, extent of bleaching, predation, competition, overgrowth, or other cause of mortality. Each coral was first carefully examined to identify cryptic predators. Lesions were initially diagnosed into four categories: recent tissue loss, skeletal damage, color change, or unusual growth patterns; an individual colony could have multiple characteristics (e.g., both color change and recent tissue loss). The location (apical, basal, or medial) and pattern of tissue loss (linear, annular, focal, multifocal, or coalescing) was recorded, and, when possible, a field name was assigned. If an outbreak of coral disease was documented, sampling of the affected corals was undertaken to further characterize the disease.

**Motile invertebrates***.* Large motile invertebrates (giant clams, large gastropods, and sea cucumbers) were recorded using roving, timed surveys within each of the dive survey sites. Motile invertebrates were also identified and counted along the coral belt transects and benthic point intercept surveys discussed above.

**Supplemental results**

**Average live coral cover (ALCC)**

ALCC was calculated across the 59 survey sites at a range of depths within each site. Over 300 benthic surveys were conducted, and the overall ALCC was 31±13% (std. dev. for this and all error terms henceforth). ALCC was statistically similar between Ha’apai (30.5±9.5%) and Vava’u (34±10%; student’s *t*-test, *p*>0.05). Emergent and submergent reefs had similar ALCC. In contrast, reefs of intermediate exposure tended to have higher ALCC (34±10%) than exposed reefs (17±12; 1-way ANOVA effect of exposure, *p*=0.06; Tukey’s honestly significant difference [HSD] of intermediate vs. exposed *p*<0.05); however, only two exposed reefs were surveyed, so these findings should be interpreted cautiously. ALCC was similar within (33±8.8%) and outside (31±11%) of the lagoon (student’s *t*-test, *p*>0.05). Finally, ALCC was similar across the five reef types: barrier forereef (33±14%; n=5), fringing reef (32±11%; n=22), patch reef (32±8.4%; n=21), barrier backreef (31±10%; n=3), and pinnacles (25%; n=1). The 12 outliers uncovered (described in the main text) were not significantly more prevalent on any reef type, nor did ALCC affect outlier frequency. For instance, outliers were just as likely to be found on a low ALCC reef as a high one.

**Site descriptions**

**Ha’apai**

*TAHA01*. This submerged, lagoonal patch reef extended from 2 to 25 m. The slope descending to 25 m was steep and ended in sand. There were prominent shallow areas (5-10 m) with 20-30% ALCC. In general, there was a high abundance of 30-70-cm table acroporids mixed with medium-sized branching acroporids, pocilloporids*,* favids (notably *Favia stelligera*), poritids (notably *Porites rus*), and others. The slope featured a high abundance of plating and columnar *P. rus* colonies and large stands of bottlebrush acroporids intermixed with *Porites lobata*, favids, medium-sized pocilloporids, *Coscinarea* spp., and other species. ALCC at mid and deeper depths ranged from 20 to 80%. In some areas, very large plating corals (50-200 cm in diameter), including *Pachyseris* sp., *Mycedium* sp., *P. rus*, and *Merulina* sp., were documented. The reef ended at 25 m in sand, and scattered, very large (2-3 m in diameter), mounding *P. lobata* colonies were observed in this area. Macroalgae were rare below 10 m. Isolated clumps of *Tydemania* sp. and 20-30-cm patches of thick, green, filamentous macroalgae (*Cladophora*-like) were documented on both the substrate and on dead corals. Shallow areas were characterized by numerous damselfish algal lawns. *Drupella* sp. snails were seen on a small number of acroporids (<1%). Several *Culcita* sp. sea stars were observed in the vicinity of small, recently eaten corals. Very little disease was present; a few isolated cases of white syndrome were documented, and a few colonies had tumors. Open areas of hard substrate had thin mats of cyanobacteria in certain places, and turf algal cover reached 30-60% in areas; such mats were typically fairly thick. Relief was high (1-3 m) with some outcrops and large boulders. Micro-relief ranged from 20 to 80 cm, with higher relief in deeper waters. Three giant clams and two species of sea cucumbers were observed (a single individual of each was seen.). Large soft corals could be found in the shallows.

*TAHO2*. The reef extended from 1-2 m to 12-15 m, then sloped very gradually to 20 m. The shallows had some large mounds and pinnacles with steeply sloping sides. There was a relatively flat terrace at 10 m that extended outwards, forming wide spurs separated by wide sand channels. The spurs were 2-3 m above the sand, and they sloped gradually into deeper waters. The shallows had large (several meter), encrusting *Favites* sp. colonies, numerous massive and columnar *Coscinarea* sp. colonies, and small- to medium-sized pocilloporids, acroporids, and *Goniastrea* sp. colonies. At 10-15 m the reef was dominated by large stands of bottlebrush coral, intermixed with favids (especially *Platygyra* sp. and *Goniastrea* sp.) and a high abundance of *Isopora* sp. Also, lower numbers of large (50-100 cm) *Merulina* sp., *Leptoria* sp., and *Galaxea* sp. were observed. A high percentage of the large *Platygyra* sp. and *Goniastrea* sp. colonies had patches of recent mortality that were colonized by turf algae. *Isopora* sp. colonies also frequently possessed white lesions that appeared to have presented themselves recently. No sea cucumbers were seen, and three large giant clams were observed. There was very little macroalgae apparent at the site, though some cyanobacteria was seen. Soft corals were common in the shallows.

*TOHA03*. Shallow, 1-3-m, flat-topped mounds separated by sand channels could be found up to 3 m below the crest of the reef, which was located inside of the channel at the edge of the reef system. As a general rule, there was high cover of soft corals, mixed with a dense and diverse assemblage of branching acroporids, pocilloporids, and small massive corals. The sides of the mounds were covered with large plating corals on the vertical surfaces, and these mounds dropped to 4-5 m, ending in sand. They were undercut with small caves and ledges, and there were numerous *Tubastrea* sp. colonies and smaller plating corals under the overhangs. The sides of the channel sloped steeply to 18 m, ending in sand. Coral cover was fairly low (5-10%). At the base there were small thickets of staghorn coral. The sandy bottom at depths was characterized by large ripples and depressions from the activity of waves and currents. *Halophila* sp. seagrass was present at low densities.

*TOHA04*. This lagoonal patch reef had a number of large mounds that extended up to 2-4 m depth; they were surrounded by sand patches at 8-10 m. The mounds had a very high abundance of table acroporids on the tops and sides that were 50-150 cm in diameter, with multiple canopy layers. These were interspersed with staghorn corals and smaller massive coral species. The table acroporid cover comprised 40-60% of the benthos, but 2-5% of these had small, white lesions from snails (possibly *Drupella* sp.) and white syndrome. There were numerous large, massive *Porites* sp. at the edges of these mounds, some 2-3-m wide and 2-m tall. Below these were sand and rubble patches, followed by low-relief, wide spurs that sloped very gradually into deeper water. The spurs had sparse coral communities with 10-15% ALCC dominated by 1) small- to medium-sized *Goniastrea* sp., 2) *Favia* sp. and other favids, 3) small- to medium-sized table acroporids and staghorn coral, 4) encrusting *Montipora* sp. up to 1 m in diameter, and 5) scattered, large *Porites* sp. colonies (0.5-1 m in diameter). At 15 m there was a band of foliaceous grey leather corals, which comprised about 5% of the benthic cover; leather corals were less common below 20 m. Many of the corals from 10 to 20 m had large patches of old partial mortality, and there were numerous damselfish lawns. The reef ended in sand/rubble at 25 m. There were patches of cyanobacteria, which comprised 5-10% cover throughout mid depths (10-20 m), and much of the substrate was covered in turf algae (40-60%). Low cover of *Tydemania* sp. (2% at 10 m), small tufts of green *Cladophora* sp., and patches of 10% cover of *Caulerpa* sp. were all observed at 15 m. Numerous *Culcita* sp. (at least five) and a moderate abundance of sea cucumbers were present.

*TOHA05*. The shallow areas of this leeward patch reef consisted of large mounds that sloped steeply from 4-5 m to 15 m, ending in a sand/rubble channel. A second reef extended seaward, and it was comprised of wide spurs that 1) sloped from the sand channel up to 12 m, then 2) sloped gradually to 15 m, and then 3) dropped fairly steeply to 25-30 m. The shallows had a high cover of branching acroporids, small table acroporids, thickets of bottlebrush acroporids, *Pocillopora* spp., and small *Montastrea* sp. and *Goniastrea* sp. colonies. ALCC was about 30%, and soft corals comprised 30-40% of the benthos in the shallows. On the edges of the mounds (near the base) there were larger plating corals intermixed with soft corals and small branching corals. The seaward spurs were fairly high relief (up to 1 m) with extensive framework, depressions, and a rugose matrix of skeletons with holes and crevices. Between 10 and 15% of the colonies were dead. Much of the complex framework was long dead, but numerous corals had colonized the skeletons. At 15 m the reef was dominated by bottlebrush *Acropora* spp., small table acroporids, some staghorn coral, small- to medium-sized *Goniastrea* sp., massive and encrusting *Montipora* sp., and moderate numbers of *Platygyra* sp., *Leptoria* sp., *Lobophyllia* sp., *Acanthastrea* sp., *Cyphastrea* sp., *Montastrea* sp., and *Coscinarea* sp. Most were 20-30 cm in diameter. The reef had numerous, recently eaten corals, especially bottlebrush acroporids. Six crown of thorns starfish (COTS; *Acanthaster plancii*) and two *Culcita* sp. pincushion starfish were spotted. Low cover (2-3%) of macroalgae (predominantly *Tydemania* sp.) and 5% cover of cyanobacteria characterized this site.

*TOHA06*. This patch reef had a series of very large mounds that extended seaward. The shoreward mound came up to 3 m and sloped steeply to 18 m; a second mound extended up to 6-8 m depth. This outer mound sloped moderately on the sides and seaward edge from 8 m to 15-23 m, then sloped more steeply to 28 m. The outer reef had an undercut ledge with *Tubastrea* sp. and some soft corals. Rubble and small corals were found in the sand at the based of the mound, which continued to slope seaward very gradually. The reef had a high cover of an encrusting/ submassive black sponge (5-10%). Soft coral declined from 10% cover in the shallows to 2% at greater depths. ALCC was low in the shallows (5%), was at its maximum from 12 to 20 m (20-30%), and then declined steeply below 23 m to < 5% at 25 m and 1% at 30 m. The shallow assemblages were dominated (non-exclusively) by *Pocillopora* spp., digitate acroporids, and medium-sized poritids. At mid depths *Goniastrea* spp. and *Montipora* spp. were dominant. On the deep reef, small *Porites* spp., *Montipora* spp., *Pocillopora* spp., *Stylophora* spp., and *Pavona maldivensis* were abundant, and most corals were 20-30 cm in diameter. Turf algae were uncommon in the shallows, but much more so at 20 m (50%), with up to 5-10% cover of macroalgae (notably *Tydemania* sp.) in deeper areas. A thin layer of sediment covered deeper reef surfaces. The reef had a fairly high abundance of sea cucumbers (>20 observed).

*TOHA07*. The shallow part of this low-relief patch reef sloped from 3-4 m to 8-10 m, ending in sand. This area had very little coral, less than 0.5 m of relief, and consisted mostly of a low cover of soft corals and extensive patches of cyanobacteria. The substrate was 8-25 m deep and was largely sand with low cover of *Halimeda* sp; there were also numerous small- to medium-sized coral bommies that were 1-3 m tall and up to 20 m wide. The coral bommies extended up to 5 m in some cases, but most came up to 8-15 m depth. Some of these formed elongate, spur-like structures that had slight build-ups on the seaward edge and a steep slope to the sandflats at 20-25 m, while others were small (2-5 m), circular mounds less than 1 m in height. These varied considerably in composition. Several were dominated by 30-50% cover of *Lobophyllia* sp. Others had a more mixed community. The deeper area had lower coral cover (10-15%), with a mix of *Goniastrea* spp., *Favites* sp., *Favia* sp., small table acroporids, *Acanthastrea* sp., *Echinopora* sp., *Montastrea* sp., *Stylophora* sp., and many other genera. Soft corals, turf algae, and cyanobacteria comprised 5-10%, 60%, and 5% of the benthic cover, respectively. At the deeper end of the reef (22 m), there were some 2-3-m-wide *Diploastrea* sp. colonies. Small stands of staghorn coral were present at the bases of the bommies. Many less common hard coral species were found, including *Euphyllia* sp., *Hydnophora* sp., and *Caulastrea* sp., and it was the first reef of the Tonga expedition where several large mounds covered by a foliaceous growth form of *Pachyseris* sp. were seen.

*TOHA08*. This reef had a shallow platform at 6-8 m, with wide, low-relief spurs intersected by shallow, narrow channels. The tops of the spurs were 0.5-1 m above the channels; these had isolated boulders that were up to 0.8 m tall, but most of the hardground was moderately low relief (up to 0.5 m), with a dominance of soft corals (especially *Lobophyton* sp., *Sinularia* sp., and *Sarcophyton* sp.). Intermixed in this community was a large number of smaller massive, submassive, and branching corals, including 1) a high diversity of rare species (e.g., *Euphyllia* sp., *Caulastrea* sp., and *Coscinarea* sp.), 2) *Pocillopora* spp., 3) small table and digitate acroporids, 4) *Goniopora* sp., and 5) *Hydnophora* sp. There was a slight build-up at the edge dominated by branching acroporids, then a moderately steep slope (vertical in places) from 12-20 m. At 20 m the slope became more gradual, continuing to 35+m. Scattered, large mounds (2-5 m in diameter) occurred on the deeper part of the reef, extending from 20-30 m in depth. Throughout the deeper part of the reef (on the slope and at the base of the slope) were extensive, 10-30-m-wide bands of monospecific assemblages. Thickets of long-branched, bottlebrush acroporids, each 20-30 m x 3-4 m, occurred in three locations. One large mound was encrusted by overlapping *Coscinarea* sp. plates, covering an area of more than 20 m x 20 m at 60-90% cover. Slightly east of these were three very large *P. rus* assemblages that featured overlapping sheets in the deepest areas, turning into plates and erect branches in shallower locales. Each of these was 5-20-m long and 5-m wide (60-90% ALCC). In another area there was a large, monospecific assemblage of *Merulina* sp., with foliaceous colonies each 1-3 m in diameter and covering 20-30 m up the slope. In another area there was a large assemblage of *Lobophyllia* sp. (5-10-m wide), which was intermixed with *Acropora* spp. in some places. The reef ended at 30 m, becoming mostly sand with some rubble.

*TOHA09*. This lagoonal patch reef featured low-relief (20-40 cm), wide spurs. These sloped moderately steeply from 3-4 m to 22 m; then, a second reef system extended from the sand up to 20 m, sloped very gradually seaward to 23 m, then dropped steeply to 30 m, and ended in sandy rubble at 31-33 m. The sand flat continued sloping seaward very gradually. The shallows at 10 m had 10-15% ALCC, which was dominated by small massive species (e.g., *Goniastrea* sp. and *Platygyra* sp.) and large numbers of small *Montipora* spp., small *Porites* sp., and *Cyphastrea* sp., amongst others. Approximately 10% of the coral was dead (in growth position). Soft corals, turf algae, and zoanthids made up 20%, 40%, and 20% of the substrate, respectively. Coral cover increased from 25% at 15 m to 30-40% at 20 m, then declined progressively to 10% at 30 m. Mid depths were dominated by *Acropora* spp., *Goniastrea* sp., *Lobophora* sp., *Astreopora* sp., and crusts of *Favia* sp. up to 1.5-m wide. Large amounts of turf algae were found throughout mid and deeper areas (60%). Cyanobacteria cover was 10% between 10-20 m, though it was not observed deeper than 25 m. At 20-30 m there were numerous table acroporids, medium-sized *Lobophyllia* sp., and both *Montipora* sp. and *Coscinarea* sp. plates (some 3-4 m in diameter) Between 2 and 5% of the table acroporids and pocilloporids showed signs of white syndrome. One *Culcita* sp. individual was found, and moderate numbers of sea cucumbers were observed between 20 and 30 m.

*TOHA10*. The shallows of this reef consisted of large mounds that sloped very gradually from 3-4 m to 6 m. They then sloped steeply on their seaward edges (near vertical) to 15 m, ending in sand. A series of wide, discontinuous spurs then sloped very gradually into deeper waters. The framework was largely dead *Porites* spp. colonies. The shallow reef was characterized by 40-60% ALCC and was dominated by table acroporids and staghorn coral. At 10 m table acroporids were mixed with high numbers of *Goniastrea* sp., massive *Porites* sp., and large *Platygyra* sp. The tabular corals made up 30% of the ALCC. About 10% of the corals were dead. Very large *P. rus* colonies (3-4 m in diameter) occurred at mid depths (15-20 m) and were intermixed with *Lobophora* sp., *Echinophyllia* sp., and other species. ALCC from 15-25 m was 10-20%, declining to 5% at 30 m. Several very large *Diploastrea* sp. colonies (3 m) occurred at 25-30 m. Also, numerous, larger *Montipora* sp. plates and crusts covered the benthos at these depths. The reef had moderately high CCA cover in the shallows (30%), though cover declined with increasing depth. Turf algae cover reached 60% at 10 m, though it also declined with depth. No macroalgae were present at the site. Soft corals were moderately abundant from 5 to 10 m (15-20%), though they became less common at increasing depths and disappeared below 20 m. There were white lesions on several of the *P. rus* and table acroporid colonies. The acroporids had the largest *Drupella* sp. snails seen to date, and such snails were also observed at the highest densities observed to date; 5% of the colonies at 5-10 m had large lesions.

*TOHA11*. This reef had a terrace consisting of a gently sloping, scoured hardground in the shallows (3-6 m), with large regions covered by turf algae. This descended to a rubble field at 10 m. A second terrace that extended seaward for about 50-100 m at 6 m depth sloped very gradually to 10 m before dropping vertically from 12 to 40 m. The reef had large, wide spurs with narrow, deep grooves. The shallows were characterized by high ALCC (40% at 5 m, increasing to 80% at 10-15 m), and the coral assemblage was dominated by small- to medium-sized acroporids (staghorn, tabular, and digitate growth forms), along with small- to medium-sized *Astreopora* sp. and *Pocillopora* spp., amongst other species. There was a slight build-up from 10 to 15 m, with 1) a high abundance of branching, bottlebrush, and table acroporids and 2) large, plating corals on the top of the slope (including *Porites* spp., *Coscinarea* sp., *Echinophyllia* sp., and *Mycedium* sp.), some 3-4 m in diameter. At 20 m there was a dominance of *Pocillopora* spp., *Pavona varians*, *Stylophora* sp., and many large *Favites* sp. and *Montipora* spp. plates and crusts. ALCC dropped to 25-30% at 25-30 m, and these depths were dominated by *Pachyseris* sp., *Montipora* spp., *Leptoseris* sp., *Mycedium* sp., *Echinopora* sp., *Echinophyllia* sp., and other plating corals. There was also 40% cover of *Caulerpa* sp., mostly on the outer horizontal faces of the wall, with corals instead in the vertical and near-vertical areas. Soft corals were most abundant at 5 m (20%), declining to 10% at 10 m and 5% from 15-20 m. There was very little soft coral from 25 m (2%) to 30 m (0%). CCA cover averaged 30-40% throughout the reef. Cyanobacteria were uncommon, though there were small patches of 5% cover at 15 m.

*TOHA12*. Large coral mounds extended from 3 to 10 m, ending in sand+rubble; then, deeper spurs ran from 8 to 15 m, sloping steeply seaward to 25 m. Shallow areas were largely scoured hardground, with small mats of encrusting red algae. ALCC was 5% at 3-5 m, and this area was dominated by 20-30-cm acroporids (of which ~30% were dead and 5% had white lesions). Soft corals (20%), *Millepora* spp. (5%), and a black encrusting sponge (5%) covered much of the substrate. At 6-10 m the reef consisted of low-relief, wide spurs with large sand channels. Rubble abundance increased with depth in these channels. At 15 m there was much more relief, with large stands of staghorn coral (up to 4-6-m wide), many of which were 60-80% dead and covered by damselfish algal lawns. CCA cover was high in the shallows (30%), though it declined to 20% at 15 m. Turf algae were uncommon in the shallows, though their presence increased with depth: 10-20% at 10 m and 20-30% at 15 m. Large numbers of damselfish lawns on dead corals were observed at both of these depths. The deeper reef from 20 to30 m was dominated by a dense coral assemblage (ALCC=~40%) consisting of 60-100-cm staghorn colonies, 80-100-cm, thick-branched *Isopora* sp. colonies, and large *P. rus* mounds. The latter ranged from 1-2 m in diameter and height. Many of these were 30-60% dead, with thick turf and many damselfish. About 2-5% of the digitate acroporids, plating *Isopora* sp., and *Pocillopora* spp. had lesions from snails. Also, numerous small corals had been recently eaten by *Culcita* sp. sea stars.

*TOHA13*. This reef featured tall, wide spurs that were separated by wide sand+rubble channels. The sides and seaward ends of these were characterized by near-vertical drops from 12 to 30 m. From 10 to 20 m, soft corals covered about 30% of the benthos, and stony coral cover declined from 10 to 5%. Hard corals were concentrated on vertical and near-vertical surfaces and included *Leptoseris* spp. (at least two species), *Pachyseris* sp., *Montipora* sp., *Mycedium* sp., *Echinopora* sp., and *Echinophyllia* sp. and *Merulina* sp. plates, with some smaller, submassive and branching corals. On more horizontal surfaces, the reef was dominated by *Goniastrea* sp., small *Galaxea* sp. colonies, table acroporids (small), encrusting *Favites* sp. colonies, *F. stelligera*, and *Pocillopora* spp. Coral cover declined from 5% at 20 m to 2% at 30 m. At 20 m the slope was near-vertical, with scattered staghorn corals, plates of *Leptoseris* sp. and *Pachyseris* sp., and several encrusting coral species. Relief was moderate (60-80 cm). The reef sloped steeply to 25 m, at which point it became gently sloping hardground that continued to 35 m. At 25 and 30 m, there was low ALCC and only 5-10 cm of relief. The few corals that did occur were encrusting *Porites* spp., isolated *Acropora* spp. tables (20-30 cm in diameter), encrusting *Montipora* sp., and *Pachyseris* sp. Turf algae covered ~70% of the bottom. CCA disease (orange) was prevalent.

*TOHA14*. This reef had a shallow terrace that sloped gradually from 4 to 8 m over about 100 m; it then sloped steeply from 10 to 25 m before sloping more gradually again. The shallows were a mix of soft corals (20-30% cover), hard coral (ALCC=50-80%), and an abundance of CCA (20-30%). At 5 m the dominant corals were staghorn thickets (1-5-m wide), digitate acroporids, small- to medium-sized table acroporids, *Pocillopora* spp., and numerous small- to medium-sized massive species. ALCC was 40-80% at the edge of the drop, with many very large *Merulina* sp. colonies (1-4 m in diameter, with erect, short blades) intermixed with branching, bottlebrush, and digitate acroporids. At 15 m the slope was dominated by 2-3 m-wide *P. rus* colonies, large *Lobophyllia* sp. colonies, table acroporids, 40-80-cm diameter staghorn colonies, columnar *Coscinarea* sp. colonies, and large plates of *Mycedium* sp., amongst other coral species. The wall also had large areas of *Millepora* sp. that comprised 20-40% cover at 15 m before declining to 10% at 20-25 m. ALCC dropped significantly below 15 m to about 10%, and the assemblage was dominated by 1) small- to medium-sized *Acropora* spp. and *Pocillopora* spp., 2) larger plates of *Mycedium* sp., *Echinophyllia* sp., and *Montipora* sp. (50-200 cm in diameter), and 3) submassive *P. lobata* colonies (up to 1 m in diameter). Turf algal, CCA, and soft coral cover was ~40, 30-40, and 10-20%, respectively. There was a large ledge at 18-25 m that was dominated by *Dendronephthya* sp. colonies. Some very large gorgonian sea fans occurred on the wall. Many of the staghorn coral thickets were covered by damselfish algal lawns.

*TOHA15*. Elongate spurs at 8-10 m depth with 1 m of relief increasing to 3-4 m of relief above sand channels at 15 m characterized this reef. The seaward ends and sides of the spurs dropped nearly vertically to 30 m depth into sand/rubble before sloping more gradually into deeper waters. This was the first site surveyed in Tonga with small clumps of *Halimeda* sp. (up to 10% cover). The spurs and surrounding sandy areas had about 10% rubble and 20% dead coral. ALCC ranged from 30-40% at 5-10 m, dropping to 20-30% at 15 m and 5-20% at 20-30 m. The shallow reef had branching and table *Acropora* spp. (50-100 cm in diameter for the latter). Thickets of staghorn coral and many of the tabular acroporids between 10 and 20 m depth had white syndrome (2-5%) and snails (2%). The deeper reef was dominated by plates of *Mycedium* sp., *Pachyseris* sp., *Echinophyllia* sp., and *Leptoseris scabra* on vertical surfaces. ALCC was lower on horizontal surfaces, and the community featured small table acroporids and massive favids, amongst other scleractinians. Soft coral cover ranged from 10 to 15%.

*TOHA16*. This leeward, lagoonal reef was a marine protected area (MPA). The shallows from 5 to 8 m were characterized by 30-50% cover of soft corals and scattered staghorns, table acroporids, and small, massive coral species; ALCC was ~10%. This hardground area dropped 2-3 m; then, the wide, discontinuous reef framework sloped gently to 30 m before ending in sand. The shallows were dominated by *Lobophyton* sp., *Sinularia* sp., and *Sarcophyton* sp., with scattered acroporids, pocilloporids, and small favids (up to 20% cover). The framework consisted of some small mounds (up to 1 m wide and 1 m tall) but mostly lower relief rocks (up to 50 cm) with scattered corals and soft corals. ALCC was low (5%) at intermediate depths (12-20 m), with some patches of higher coral abundance (up to 25% ALCC) and 10-20% cover of soft corals. Below 15 m the dominant soft coral was the thin, flattened, grey leather coral; it was less common below 25 m. The deeper part of the slope had some medium to large, plating *Echinophyllia* sp., *Pachyseris* sp., *L. scabra* (50-200 cm), *Montipora* sp. (many with partial mortality), medium-sized *Goniastrea retiformis* and *Lobophyllia* sp. colonies (50-100 cm), medium-sized massive *Porites* spp., *Favia* sp. (50-100 cm), and numerous fungids. Throughout the reef there were patches of cyanobacteria (0.5-1 m in diameter), some turf algae, and no macroalgae. CCA were prominent in the shallows (to 12 m); fine silt covered the bottom in deeper areas, which also had an abundance of green rope sponges. There was a moderate abundance of sea cucumbers. Four *Culcita* sp. seastars were observed, as were five COTS.

*TOHA17*. This leeward forereef site was only dived at night and so was not surveyed to the extent that the other sites were. It was clear that the reef had high abundance of coral from 5-15 m; however, there was little coral deeper than this. The 5 to 15 m area was dominated by small- to medium-sized acroporids and pocilloporids, with 30-40% cover of soft corals.

*TOHA18*. This leeward forereef was characterized by large mounds with flat tops at 6-8 m and steeply sloping sides extending to 15 m; then, lower relief spurs sloped gently to 35 m, ending in sand and rubble. The shallow areas had large stands (some up to 10 m long) of staghorn and shorter-branched acroporids intermixed with *Pocillopora* spp., other acroporids, and many massive species. From 10 to 20 m depth, there was a large number of very large table acroporids (notably *A. cytherea*; some up to 3 m wide) intermixed with staghorn corals. ALCC from 5 to 20 m was 50-80%, with 10-20% soft coral cover in the shallows; few soft corals were seen in deeper areas. At 20-30 m ALCC dropped to 30-40%, with a dominance of large *Lobophyllia* sp. (up to 1.5 m wide), intermixed with massive species, plating *Montipora* spp., *Echinophyllia* sp., *G. retiformis*, and table and staghorn acroporids, amongst other species. The sides and bases of the spurs from 15 to 30 m had some stands of staghorn corals, but many of these were dead or partially dead areas colonized by damselfish. About 5% of the table and staghorn acroporids were dead in growth position, and 2-3% had white syndrome or *Drupella* sp. damage. No macroalgae or turf algae were found deeper, though CCA were prevalent in the shallows. When turf was found, it was mainly in the vicinity of damselfish. No COTS or *Culcita* sp. seastars were seen at this site, and *Drupella* sp. appeared on <1% of corals.

*TOHA19*. This forereef was near the east side of the island. The shallows had large mounds that were 1-3 m below the surface and sloped steeply on the sides and seaward edges to 10-15 m. The tops of the mounds had moderately low ALCC (10-20%), with a large number of damselfish algal lawns in staghorn thickets. Approximately 20% soft coral cover (namely *Lobophyton* sp., *Sinularia* sp., and *Sarcophyton* sp.) characterized this area. The sides of the mounds had patches of up to 2-m diameter plating corals (including *Pachyseris* sp. and *P.* *rus*), some larger massive *F. stelligera*, *Pocillopora* spp., and various acroporids. Very wide, low-relief (< 1 m above the sand) spurs gradually sloped to deeper waters. There were occasional, larger frameworks (up to 1 m tall), but most of the spurs had small- to medium-sized corals and dead skeletons up to about 50 cm tall. ALCC was relatively low (10-20%). There were large numbers of medium-sized, dead acroporids (5-10%) and many corals with partial mortality. Spurs were dominated by *Goniastrea* sp., small table acroporids, staghorn coral, *Montipora* spp. crusts, *Stylophora* sp., small *Montastrea* sp., *Favia* sp., other favids, and *Pocillopora* spp. Additional plating coral species were found deeper. Much of the hardground was covered by turf algae on CCA, with patches of cyanobacteria. There were moderate numbers of sea cucumbers; four species were observed in sandy areas, notably the small, spiky, black species (at 8-10 m depth). No macroalgae were present, though some small patches of *Halimeda* sp. were found at the bases of the ledge at 10-12 m depth.

*TOHA20*. The shallows (2-5 m) of this leeward lagoonal reef in the special protected area (just outside of the aforementioned MPA) were dominated by 1) large *Lobophyton* sp. and *Sinularia* sp. colonies (60% cover), 2) some tabular, thick tabular, branching, and digitate acroporids, and 3) small massive corals. Many of the acroporids had snail damage (5%), and snails were observed on both staghorn and tabular acroporids. Corals with mortality were also covered by numerous damselfish lawns. Occasionally, 1-2-m patches of reef framework possessed thick crusts of CCA. From 5 to 10 m there was mixed reef framework interspersed with patches of sand. In places the framework was up to 1 m tall with scattered hard corals and 20-30% cover of soft corals. Corals included small- to medium-sized favids (20-30 cm; *Favia* sp., *Goniastrea* sp., *Favites* sp., and *Platygyra* sp.), tabular and branching acroporids (most < 50 cm), and fewer *Pocillopora* spp., *Seriatopora* spp., *Stylophora* sp., *Lobophyllia* sp., *Astreopora* sp., and *Euphyllia* sp., amongst others. At 10-15 m there were low relief spurs (up to 50 cm tall) with some larger patches of framework and dead boulders with 5-10% ALCC, 30-40% dead coral cover, and 10% soft coral cover; more than 50% of the benthos was sand with scattered rubble in most places. Below 15 m there was only 5% ALCC and 5% soft coral cover (mostly *Lobophyton* sp.). Corals included isolated, medium-sized, massive *Porites* (up to 1 m in diameter), *Montipora* spp. crusts, favids, small acroporids, and a number of plating corals; the latter included *Mycedium* sp., *Echinopora* sp., *Pachyseris* sp., *Merulina* sp., and *Goniopora* sp., most of which were less than 1 m in diameter. Live coral ended at 20 m in the sand. Sand with small corals, dead corals, and some rubble sloped moderately steeply into deeper waters. Many of the corals were characterized by partial mortality. No macroalgae were present, though CCA were seen on rocks to 15 m. Turf algae were mainly associated with damselfish lawns. There was a moderate abundance of sea cucumbers (three species) in the sand and adjacent to corals. Giant clams were rare, and three and four *Culcita* sp. and COTS were observed, respectively. *Drupella* sp. snails were present, and white syndrome was observed on some acroporids. The site was inhabited by very few fish.

*TOHA21*. At this outer, leeward forereef, an uplifted coral island sloped down to 3-5 m vertically before extending out about 100 m on a terrace that was 5-7 m deep. The terrace was a scoured hardground in places and featured scoured sand channels (with minimal sand and some rubble) and scattered, large boulders (3-5 m in diameter) that had fallen into the sea from the shore. The sides of the boulders were colonized by hard corals (mostly *Pocillopora* spp. and *Acropora* spp.) and soft corals. The reef substrate had very little coral on the vertical slope near shore and the horizontal scoured hardground for the first 50 m; mostly small *Pocillopora* spp. and *Acropora* spp. colonies, as well as some encrusting poritids, were all that were observed there. Colonies were low-lying (10-20 cm tall) and ALCC was only 2%. Seaward of the terrace there were 1) hardground areas with turf algae, 2) CCA-encrusted substrate, and 3) scattered corals (ALCC=5-10%); the latter group included mostly 1) small- to medium-sized (15-25 cm), digitate, and tabular *Acropora* spp., 2) *Pocillopora* spp., 3) *Astreopora* sp., 4) *Montipora* sp., 5) submassive *Porites* spp., and 6) large numbers of small *Montastrea* spp. (10-15 cm). Soft corals were also common; *Sinularia* sp., *Sarcophyton* sp., and *Lobophyton* sp. comprised 20-50% of the benthic cover. This was the first reef characterized by large mats of *Briareum* sp. encrusting the substrate and overgrowing corals. There was about 5% cyanobacteria cover on the terrace but no macroalgae. The edge of the slope (from 7 to 10 m) was high relief (1-2 m), with prominent channels and a slight build-up dominated by thick-branched *Isopora* spp. (many 80-100 cm). The edge was intermixed with encrusting and branching *Millepora* spp., *F. stelligera*, and lower abundances of *Goniastrea* sp., *Pocillopora* spp., and *Astreopora* sp. The wall had medium to large plating and submassive poritids (50-200 cm), plating *Goniastrea* spp., *Isopora* spp., *Montipora* spp. plates, sheeting *P. rus* (up to 2-m high shingles), smaller *Montastrea* spp., *Pocillopora* spp., and other species. Large numbers of *Pachyseris* sp., *L. scabra*, *Echinophyllia* spp., and *Echinopora* sp. plates were observed between 20 and 30 m. Soft corals comprised 10-20% of the benthic cover on the wall. There was good colonization by CCA and abundant turf algae. From 8 to 15 m depth, high numbers of damselfish algal lawns were observed, especially within *Isopora* spp. colonies and staghorn coral thickets. No sea cucumbers were seen, and there were low numbers of giant clams.

*TOHA22*. The shallows of this fringing reef, which abutted another island in the north of the archipelago, had a terrace at 4-8 m depth, with scoured channels and about 0.5 m relief. This area had fairly low ALCC (10-20%); there were mostly small table and digitate acroporids, *Pocillopora* spp., *Astreopora* spp., small *Porites* sp., large numbers of small *Montastrea* sp. colonies, and large numbers of soft corals (30-40% cover). Exposed reef surfaces had good cover of CCA. The near vertical wall sloped to 40+ m with deep, narrow channels. Much of the framework on the wall was thick-branched corals. The shallow part of the slope had small- to medium-sized table acroporids, staghorn coral, *Montipora* spp. plates, and large patches of encrusting *Millepora* sp. The deep reef (18-40 m) had 60-90% cover of *Caulerpa* sp. (higher below 20 m). Interspersed were patches containing mostly plating corals on vertical surfaces, including *Echinophyllia* sp., *Pachyseris* sp., *L. scabra*, *Mycedium* sp., and *Montipora* spp. The entire wall had very high relief, with small ledges, crevices, and depressions that were up to 1-2-m wide and 1-m deep. The sides of these structures tended to have the highest ALCC, while *Caulerpa* sp. was densest on the exposed, outer surfaces of the wall. At 40 m there was a small terrace; this was followed by a more gradual, sandy slope. A second species of *Caulerpa*, as well as small clumps of *Halimeda* sp., were found here.

*TOHA23*. The landward side of this fringing reef had mounds that sloped gradually from just below the surface to a sand flat at 10 m. A second reef system extended seaward, sloping up from the sand to 5-7 m, then dropping steeply to 40+ m. The back side of this reef had large stands of thick-branched staghorn acroporids mixed with branching *Isopora* spp., small table acroporids, and bottlebrush acroporids. The slope was near vertical in hardground areas, but sand/rubble channels interweaved through these hardground areas. The top of the reef was dominated by dense stands of bottlebrush and short-branched staghorn acroporids intermixed with other corals. Within holes and crevices between the acroporids were small *Galaxea* sp., *Pocillopora* spp., *Coscinarea* sp., *Caulastrea* sp., *Astreopora* sp., *Leptoseris* spp., and other corals, as well as patches of false corals. The acroporid thickets extended to about 15 m depth, being most dense at the top of the reef and dropping off to 5-10% cover by 15 m. These acroporids also had considerable old mortality on the slope, though less so on the top of the reef. Below this was a band of branching *Millepora* sp. from 15 to 20 m. ALCC was lower deeper (5-10%), and the assemblage was dominated by plating *Montipora* spp., *Echinophyllia* sp., *Echinopora* sp., *Goniastrea* sp., *Pachyseris* sp., submassive *Porites* spp., *Stylophora* sp., and small table acroporids. Deep areas had few leather corals but higher numbers of gorgonian sea fans. There were many areas on the reef slope with dead or partially dead staghorn and table acroporids with dense turf algae and interspersed patches of cyanobacteria. Fused rubble on the slope had high cover of CCA, though such CCA were covered in cyanobacteria, some macroalgae (*Tydemania* sp.), and turf in places. This was the first reef in Tonga where *Lobophora* sp. (brown algae) was seen. There were about two dozen areas with aggregates of small, COTS-eaten corals. Twelve and one COTS and *Culcita* sp. were seen, respectively, as were a number of larger giant clams.

*TOHA24*. The shallow, seaward portion of the crest (2-4 m) of this windward fringing reef was scoured hardground, with 5-15 cm of relief and 1-2-m deep, scoured channels. At 5 m there were small, 5-15-cm, low-relief corals (< 2% ALCC), as well as 5% cover of soft corals, 5% *Palythoa* sp. cover, small tufts of *Halimeda* sp., erect CCA, and scoured CCA. Slightly deeper the CCA were covered in turf algae (60%). The reef sloped gradually to 8 m, then more steeply to 30 m. ALCC was 10% at the edge of the drop (10 m), and the assemblage was comprised mostly of small *Montastrea* spp., massive *Montipora faveolata*, *Astreopora* sp., thick plating and tabular *Acropora* spp., and *Platygyra* sp., amongst others. Most corals were 10-20 cm in diameter and no more than 15 cm tall. The bottom had good CCA cover, and there were numerous, 10-20-cm tall CCA spires. This area also had 30-40% cover of soft corals, 10-20% cover of encrusting *Millepora* sp., and 10% cover of *Palythoa* sp. At 15 m ALCC was 10-20%, and the assemblage was dominated by 1) very large plates of *Montipora* sp. (one was 6 m across.), 2) thick, table *Acropora* sp. colonies, 3) staghorn and digitate *Acropora* spp., and 4) 10% cover of *Millepora* sp. There were numerous damselfish lawns and up to 60% turf algal cover. The reef was low relief (10-20 cm). At 20-25 m there was 15-20% ALCC, with a dominance of *Isopora* sp. plates, *Lobophyllia* sp., *Montipora* sp., *Mycedium* sp., *Astreopora* sp., large patches of turf algae with damselfish amongst dead corals, and 30% cover of soft corals. ALCC declined to 15% at 25-30 m, with some medium to large *Porites* sp. colonies (50-80 cm; mostly submassive), large plates of *Isopora* sp., *Pachyseris* sp., *Mycedium* sp., and other plating species. There was 20% cover of *Halimeda* sp. and 20-25% soft coral cover. The deeper areas had 50-80 cm of relief.

*TOHA25*. The shallows of this windward, fringing forereef near the reef crest was scoured hardground with some deep, narrow channels. From 3 to 9 m there was very little relief (10-20 cm) or coral (<1-2% ALCC). The substrate had good colonization of CCA mixed with patches of turf algae, *Halimeda* sp., and macroalgae. At 8-15 m cover of *Microdictyon* sp. (algae) ranged from 10 to 30%. *Halimeda* sp. cover was 10-20%, and scattered patches of cyanobacteria were also present. The reef sloped very gradually to 10-12 m; then, there was a second terrace sloping up slightly and then gradually down to 12-15 m before dropping more steeply. The top surface of the deeper terrace was largely flat, with some encrusting and submassive corals and large areas of thick turf with damselfish. The deeper mounds were also low relief; these started at about 15 m and sloped gradually seaward to 25 m. They then dropped more steeply. On these deeper mounds, the dominant coral was a grey, thin, foliaceous leather coral. Throughout the reef were small patches of 5-10% coral mixed with 5-10% cover of soft corals. Most corals were low-relief and small (5-15 cm), with isolated, larger *Montipora* spp., *Isopora* sp. plates, submassive poritids, and *Astreopora* sp. Many of the *Astreopora* sp. colonies had small tufts of macroalgae and cyanobacteria on their surfaces. A large number of snails were found on *Isopora* sp., *Montipora* sp., and *Acropora* sp. colonies. One *Culcita* sp. individual and one COTS were seen, though neither sea cucumbers nor giant clams were observed.

*TOHA26*. The shallow areas of this lagoonal fringing reef consisted of large mounds that extended from 3-4 m to 14 m, sloping steeply to a sand/rubble field. A second set of very wide spurs extended seaward, sloping very slowly to 25 m, then dropping steeply to 35 m before ending in sand. The sandy areas between the spurs were 0.5 m below the reef framework in the shallows, increasing to 1-2 m deeper. On the shallow reef there was an abundance of 1) *Isopora* sp. spires, 2) digitate, small, tabular, and thickly branched acroporids, 3) *Pocillopora* spp., 4) small- to medium-sized favids, and 5) *Lobophyllia* sp., amongst other species. There were also several 2-3-m diameter *Diploastrea* sp. colonies at the bases of the shoreward mounds. The reef framework from 10 to 25 m was complex and featured 60-150 cm of relief consisting of 1) large, very eroded, dead massive corals, 2) dead table acroporids, 3) thick, fused staghorn skeletons, and 4) other corals, with numerous crevices, depressions, and small holes that extended into the framework. These corals appeared to have died 5-10 years ago and were heavily colonized with many species of massive, plating, and branching corals. Most massive coral colonies were only 2-15 cm in diameter. Plates occurred on the sides and between the upright framework. Large areas of bottlebrush acroporids were found in this area, mostly with just the top 10-15 cm still alive. This area was in the midst of a minor outbreak of COTS at the time of surveying. COTS were observed in small groups; most were hidden deeply in holes and under the coral framework, but each had 5-10 recently eaten corals near their dens, and there were many corals with 100% transitional mortality. In many cases, they ate the acroporids on the top of the reef and also the encrusting and massive corals in the depressions near their dens. Seven COTS were collected and five additional starfish were seen. The reef had 2-5% cover of *Tydemania* sp. algae, 30-60% turf algal cover, and 30-40% CCA cover. Soft corals were rare (2-5%) except shallower than 8 m.

*TOHA27*. Located well offshore, this submerged, lagoonal patch reef crested 3-4 m below the surface. In the shallows there were a number of very long (>200 m), wide (20-30 m) spurs that extended from 7-8 m to 15 m in sand/rubble channels. The edges of these spurs sloped steeply from 12-15 m to 25 m, ending in sand, with scattered, small coral bommies (< 1 m) that continued to slope gradually into deeper waters. The tops of the spurs (ALCC=20-30%) were dominated by bottlebrush, staghorn, digitate, and small table acroporids, *Goniastrea* sp., *Favia* sp., *Platygyra* sp., and *Astreopora* sp. (most 20-40 cm in diameter). The spurs away from the reef’s edge had assemblages comprised of *Goniastrea* sp., medium to large, columnar *F. stelligera*, scattered larger *Porites* sp. (1-2 m in diameter, though with some very large [2-3 m] colonies in the channels between the spurs), and a high number of 1-2-m plates of *Turbinaria* sp. (most with partial mortality). *Pocillopora* spp., *Coscinarea* sp., *Lobophyllia* sp., *Montastrea* sp., and *Acanthastrea* sp. were also found in this area, amongst other taxa. There were also numerous, medium-sized *P. clavus* colonies. Soft coral cover ranged from 5-20% at 5-10 m depth, 25-30% at 10-15 m, and ~0% over the edge. The sides of the slope from 12 to 20 m were dominated by 1) 30-40-cm *Goniastrea* sp. colonies, 2) 20-30-cm pillars of *Isopora* sp., 3) *Porites* spp., 4) *Montipora* spp., 5) *F. stelligera*, 6) *Coscinarea* sp., and 7) *Pocillopora* spp., amongst others. Approximately 10% of the staghorn corals from 15 to 25 m depth were dead or mostly dead and coated by thick turf algae and damselfish. At the base of the slope there were 10-20-m long thickets of staghorns, of which ~30% were dead and covered by damselfish turf algal lawns. The slope had 5-10% cover of encrusting and submassive black sponges, 5-10% cover of *Lobophyllia* sp., 20-30% cover of *Peyssonnelia* sp., patches up to 5 m of *Tydemania* sp., and 30% cover of turf algae, which increased in deeper areas (20-25 m) to 60%. The sand flat extending away from the reef had 0.5-2-m diameter boulders plus some larger areas of framework up to 10-m long that were 30-100 cm above the sediment. This area was characterized by 10-30% ALCC, and the assemblage was comprised of mostly small colonies, including *Goniastrea* sp., *Lobophyllia* sp., *Hydnophora* sp., *Pocillopora* spp., small acroporids, *Favia* spp., *Favites* spp., and many other hard coral species. Turf algal cover was 50-60%. Three *Culcita* sp. seastars were seen, and several acroporids and pocilloporids were being preyed upon by snails.

*TOHA28*. This lagoonal patch reef was characterized by 1) several shallow, emergent mounds (1-2 m deep) and 2) gently sloping spurs. The shallow areas (3-5 m) were largely scoured hardground with dense turf algae, though isolated corals were also present. At the edge of the mounds was a dense, *Acropora* spp.-dominated community (mostly digitate, though with some thick tables and staghorns), with massive *Montipora foveolata*, *Pocillopora* spp., *Coscinarea* sp., *F. stelligera*, *Favites abdita* (crusts), *Pachyseris* sp., *Gardinoseris* sp., and many others. ALCC was 50-80% while soft coral cover was 5-20%. The mounds sloped steeply to 10-15 m, and there were large, columnar *F. stelligera* colonies, thick-branched and digitate acroporids, *Pocillopora* spp., *Coscinarea* sp., *Platygyra* sp., plates of *Echinopora* sp. and *Echinophyllia* sp., and other species on the vertical or near vertical sides. The mounds had an undercut ledge at the base with some *Pachyseris* sp., *Leptoseris* sp., and other species. ALCC was 5-10%, and *Peyssonnellia* sp. carpets comprised much of this. At the base there was a series of wide sand patches interspersed with large rubble fields. Adjacent to these were long, wide spurs. These sloped up to 12-13 m at the top before sloping very gradually seaward. In deeper areas, there was a 3-4-m distance from the top of the spur to the sand channel. The spurs were dominated by diverse assemblages, including 1) bottlebrush acroporids, 2) *Acropora* spp. mixed with small- to medium-sized massive species (especially *Goniastrea* sp. and *F. stelligera*), 3) mixed communities of favids, *Astreopora* sp., acroporids, *Pocillopora* spp., *Lobophyllia* sp., and other species, and 4) staghorn thickets. The staghorn thickets were 30-40% dead with numerous damselfish in their vicinity. There were no large table acroporids. ALCC ranged from 5 to 30%, with dense *Lobophora* sp. encroaching on the bases of the coral framework. Open areas were covered in *Peyssonnelia* sp., with patches of *Tydemania* sp., cyanobacteria, and other algae. Many damselfish lawns were present in the staghorn acroporid-dominated areas, and numerous *Goniastrea* sp. colonies had large, multifocal lesions. There was also considerable damage at 20-23 m from COTS, two of which were collected.

*TOHA29*. Large spurs extending off an emergent island characterized this lagoonal patch reef. These ran from 3-4 m to 20 m; others were from 6-8 m to 20 m. They were 20-50 m in width and 100+ m long and were separated by wide channels filled with rubble. The spurs ended at 18-22 m in a large, sandy rubble field. At the bases of some of the spurs and mounds closest to the island, there was an undercut ledge with low ALCC. The deeper spurs sloped steeply from 10-12 m to 20 m, ending in sand. The shallow reef community was dominated by thick table acroporids, digitate, bottlebrush, and staghorn acroporids, small- to medium-sized *Pocillopora* spp. colonies, and small- to medium-sized massive species colonies (including *F. stelligera*, *P. clavus*, *Goniastrea* sp., *Lobophyllia* sp., and *Montipora* spp., amongst others). Some areas had 50-90% cover of soft corals. Several *Lobophyton* sp. colonies were 6-7 m wide, carpeting dead substrate and overgrowing hard corals. *Pocillopora* spp. tended to avoid being overgrown, but massive favids were readily overgrown. ALCC ranged from upwards of 50-60% at 5-10 m depth to areas with 10-20% ALCC and 60% cover of soft corals. On the sides were small thickets of thick staghorn and bottlebrush acroporids, *Coscinarea* sp. columns, *Goniastrea* sp., and medium-sized *Porites* sp. colonies. The more vertical areas were characterized by assemblages of *Echinopora* sp., *Pachyseris* sp., *Montipora* sp., large *Mycedium* sp. plates, *Isopora* sp. crusts, medium-sized *Pocillopora* spp. colonies, and other species. *Halimeda* sp. cover increased from 5% at 10 m depth to 20% at 20 m. In the sand/rubble fields there was some low-relief framework with 5-10% ALCC; this assemblage was mainly comprised of mostly small (10-20 cm) favids, *Lobophyllia* sp., *Symphyllia* sp., and other species.

*TOHA30*. This was a leeward barrier forereef off northeastern Ha’apai. Shallow mounds extended out from 3-4 m to 10 m, then dropped vertically to 30-40 m, ending in a sand/rubble/coral field that continued to slope gently seaward. A number of deep channels separated each mound. The tops of the mounds were scoured hardground near the reef crest, with moderately high cover (30-40%) of staghorn, digitate, thick plating, and tabular acroporids (some over 2 m in diameter), *Pocillopora* spp., *F. stelligera*, *Coscinarea* sp., *Leptoria* sp., *Goniastrea* sp., *Lobophyllia* sp., large *Favites* sp. crusts, and many other species. Soft coral cover was 25-30% near the edge of the drop-off. The mounds dropped near vertically, with small ledges, overhangs, and crevices. The 10 m area was mostly comprised of *Montipora* spp., digitate and staghorn acroporids, medium-sized *Porites* sp. colonies, *Pocillopora* spp., *F. stelligera* columns, and *Favites* sp. crusts. At 15 m plates of *Isopora* sp., staghorn and digitate acroporids, *Porites* sp., and *F. stelligera* dominated (ALCC=30-50%). There was 80-100 cm of relief on the vertical surfaces, where ALCC ranged from 5 to 50%; these assemblages consisted of plating *Montipora* spp., *submassive* Porites sp., small acroporids and pocilloporids, *Goniastrea* sp., and other species. Deeper areas were dominated by numerous sheets of *Mycedium* sp., *Merulina* sp., *Pachyseris* sp., *Leptoseris* sp., *Echinophyllia* sp., and other species. At the base of the wall (35 m) there were very large, foliaceous colonies of *Mycedium* sp., *Pachyseris* sp., *Merulina* sp., *Leptoseris* sp., and *Montipora* spp. Further south there was a steep slope (45-60 degrees) with 80-100% cover of plating and encrusting corals extending from 20 to 32 m. This assemblage included 3-5-m encrusting *P. lobata*, *P. rus*, *Montipora* spp., and other species. Further along there was a large stand of thick-branched staghorn coral, with mounds of *P. rus*, *Montipora* spp., *Goniastrea* sp., and many other species. The deep part of the wall had many 1-2-m gorgonian sea fans. Soft coral cover decreased with depth: 30% at 8 m and 5% at 30 m. Several predatory snails were seen on smaller staghorn acroporids. Parts of the wall had 10-20% cover of *Millepora* sp., and large groups of corallimorphs were seen at the edge.

*TOHA31*. At this fringing reef off the north side of the island, there was a series of very large mounds (some 3-4 m from the surface) and other seaward mounds coming up to 4-5 m beneath the surface. The tops of the mounds ranged from low cover, scoured, turf-colonized hardground away from the edge, to dense coral and soft coral communities at the edges of the slope. The mounds sloped moderately steeply on the sides and seaward end to sand/rubble channels and a sand field at 30 m. Channels were mainly comprised of sand with a significant amount of rubble, along with scattered, large (1-3 m in diameter), mounding *P. lobata* colonies. The tops of the mounds were dominated by digitate acroporids, *Astreopora* sp., *Pocillopora* spp., *Montipora* spp. plates, small massive colonies, *Goniastrea* sp., large (up to 1 m in diameter), massive *Turbinaria peltata*, and many other smaller corals. Soft coral cover was 20%. The sides had more *Porites* spp., *Coscinarea* sp., *F. stelligera* columns, *Goniastrea* sp., *Astreopora* sp. mixed with plating *Montipora* spp., *Isopora* sp., small table acroporids, and other species. The degree of partial and whole-colony mortality increased with depth, with ALCC cover declining from 20% in the shallower regions to 5% at 30 m. Massive favids (especially *Goniastrea* sp., *F. stelligera*, *Favia favus*, *Platygyra* sp., and crusts of *Favites* spp.), *Stylophora* sp., *Porites* spp., *Leptoria* sp., and *Isopora* spp. occurred on the deeper reef, but many of these were dead. Recent damage from snails was common on acroporids, in particular. Many *F. stelligera* colonies, as well as those of a few other species, were partially bleached. Turf algal cover increased from low (5-10% on the tops of the mounds) to 60-80%, with dense carpets in damselfish territories. Two *Culcita* sp. seastars were seen in the vicinity of many small, dead corals on the tops of the mounds. Several sea cucumbers were observed in the sand channels.

*TOHA32*. This fringing reef off the northern tip of the island had a shallow terrace at 4-5 m that was scoured in the center. There was an acroporid+pocilloporid-dominated community at the edge, with 20% soft coral cover and up to 30% cover of corallimorphs. The sides were steeply sloping from 8 to 18 m depth, with a mix of small- to medium-sized colonies of staghorn coral, table acroporids, *Pocillopora* spp., massive *Porites* sp., *Stylophora* sp., *Coscinarea* sp., and other species. On near vertical surfaces there was 10-20% cover of *Halimeda* sp. and 2-5% cover of *Cladophora* sp. tufts. The sides had 20-30% cover of corallimorphs, and corallimorph carpets spanned 22-25 m in places. At 15-20 m there was an increase in the number of plates and crusts of *Montipora* spp., *Isopora* sp., *Pachyseris* sp., *Mycedium* sp., and large, columnar *Coscinarea* sp. colonies, amongst other species.

**Vava’u**

Certain surveyed sites were tourist dive sites, in which case the tourist site name follows the KSLOF site code.

*TOVA33*. “Serene.” This fringing reef in the lagoon off a small island had a large terrace that extended from the island at 1 m depth; it then dropped into a sand flat at 8 m before rising up to a second terrace that extended seaward. This second terrace sloped steeply to 20 m and then more gradually into deeper waters. The top of the reef at 3-5 m had a dense colonization of stony corals mixed with soft corals.

*TOVA34*. “Pete’s Paradise.” This fringing reef in the leeward part on the lagoon had a shallow reef flat community running parallel to land that was broken up by narrow, deep channels. The edge of this structure was at 4-8 m, where it sloped at 45 degrees to 15-25 m. There were some very wide spurs that had 80-100 cm of micro-relief with extensive *Acropora* spp. build-ups. These ended in a relatively flat sand bottom area with large mounds of staghorn corals, bottlebrush acroporids, scattered tabular acroporids, and large, foliaceous plating colonies. Further seaward (20-40 m) there were additional large mounds that were up to 5 m tall, and these were dominated by huge stands of bottlebrush and staghorn acroporids mixed with patches of large *Echinophyllia* sp., *Echinopora* sp., *Merulina* sp., *Leptoseris* sp., and *Pachyseris* sp. plates. ALCC ranged from 20 to 80% throughout the site. In general, the sides and deep areas had high ALCC; however, many of the staghorn colonies had large, dead, turf-encrusted branches due to damselfish “gardening.” The dead areas were usually up to 1 m wide, with multiple gardens in any given area.

*TOVA35*. “Aquarium.” This lagoonal patch reef had a shallow, mostly flat, 2-4-m deep terrace that consisted of scoured hardground in places and soft corals and a mixed branching coral community in others. Off the southeastern end at 6-12 m there was an extensive *Porites* spp.-constructed reef, with some colonies up to 2-5 m in diameter and up to 3 m tall. These were surrounded by large staghorn coral thickets. The rest of the patch had a moderately steeply sloping community. On the north side, the reef sloped moderately from 5 to 15-18 m, then gradually to 20-23 m before ending in sand. There were numerous table acroporids and staghorn clumps running down the reef from 5-10 m depth. At mid depths there was a mix of massive corals, encrusting *Favites* spp., high numbers of crustose *Isopora* sp., table acroporids, *Merulina* sp., *Goniastrea* sp., and other species. Relief was 40-60 cm, and there were small patches of rubble and large, dead patches with damselfish algal lawns (20-60% turf algal cover). At 20 m there were 1) 1-2-m patches of *Pachyseris rugosa*, 2) 20-60-cm diameter table acroporids, 3) columnar *Isopora* sp. colonies, 4) medium-sized *F. stelligera* colonies, 5) a high abundance of *Stylophora* sp., 6) small *Lobophora* sp. colonies, 7) *Mycedium* sp., and 8) *Leptoseris* sp. and *Pachyseris* sp. plates. ALCC was 20-40%, and turf algal, CCA, and *Peyssonnelia* sp. cover were 30, 20, and 20%, respectively. There was an abundance of rubble in small sand patches between the corals. The sand flat at the base of the reef had some larger table acroporids at a low density.

*TOVA36*. “Split Rock.” This was a fringing reef on the seaward edge of a small island at the entrance to the channel. The island had a vertical face that dropped to 4-8 m. Seaward of this were numerous, large, scattered boulders (3-5 m across and up to 4 m tall) at 8-10 m depth, with sand and rubble surrounding these. There were some gorgonians, soft corals, and a few acroporids and pocilloporids on these boulders, but ALCC was generally very low. Seaward of these there were wide spurs with channels. Both the tops and sides of the spurs were dominated by large, mounding *P. lobata* colonies (ALCC=60-90% cover in most locations). The colonies were largest and most abundant from 10-15 m, declining slightly in size and becoming intermixed with other corals in deeper water. Most colonies were 50-400 cm in diameter and up to 1.5 m tall. Dead areas and the substrate surrounding the colonies were colonized by scattered tabular and digitate acroporids, pocilloporids, *Coscinarea* sp., *F. stelligera*, and large *P. rus* colonies. At the deeper end of the reef, there were also large stands of *Turbinaria retiformis*, *Stylophora* sp., and other species intermixed with the *P. lobata* colonies. The turbinariids had extensive patches of recent mortality. There were also many locations with damselfish algal lawns, including on many of the *P. lobata* colonies. In these areas entire colonies and parts of colonies had white lesions from damselfish bites, with portions colonized by thick turf algae. Throughout the reef 2-5% of the acroporids and pocilloporids had partial mortality and white areas recently eaten by *Drupella* sp. snails.

*TOVA37*. “Sea Fan.” This site was a leeward fringing reef on the back side of a small island at the entrance to the channel. In the shallows there was a vertical drop from the cliffs to 4-5 m, then a relatively flat terrace from 4 to 7 m; this was followed by a near vertical drop to 8 m, then a steeply sloping system of spurs. There was also a second platform that came up to 4 m and was separated from the island by a channel at 8-12 m depth. This structure was flat on top, with very few corals; the corals that were there tended to have snail predation damage or partial mortality that had been converted into damselfish algal lawns. The terrace closest to the island had little coral near the island itself (mostly a scoured hardground with CCA and some turf algae); ALCC was 10-20% at the edge, and the community was dominated by 1) small *Porites* spp., 2) *Montipora* spp., 3) small, digitate, and tabular acroporids, 4) *Pocillopora* spp., 5) *Coscinarea* sp., and 6) other small corals. Soft coral cover was 10-20%. There was a channel at the base of the terrace; it was mostly hardground structure with rubble. In the vicinity there was a large cave (5-m tall) with two openings, and numerous gorgonians surrounded the cave. Inside was an area with numerous small soft corals, hydrozoans, and small sponges on the ceiling. A second cave had black coral at the entrance. The shallow part of the slope to 15 m had extensive areas of rubble and large, broken plating corals in piles; most were dead. There were also several larger (3-4 m in diameter) *P. rus* colonies. Below 15 m ALCC was 20-30%, declining to 10% at 25 m and 2% at 30 m. This part of the reef was dominated by 1) *P. lobata*, 2) *Montipora* spp. crusts, 3) *Lobophyllia* sp., 4) small *Goniastrea* sp., 5) *Astreopora* sp., and 6) small- to medium-sized branching acroporids, pocilloporids, and stylophorids. Below 15 m about 10% of the benthos was covered in an encrusting/submassive black sponge. Numerous *Drupella* sp. lesions were seen on branching corals, and there were large numbers of damselfish algal lawns. Finally, several *Culcita* sp. seastars were seen.

*TOVA38*. “North shore.” The reef platform had a terrace at 4-6 m dominated by small- to medium-sized digitate and table acroporids, *Pocillopora* spp., *Astreopora* sp. (5-10% ALCC across all species), and large areas of hardground; the latter areas tended to have thick turf and damselfish lawns. The terrace dropped near vertically to 8-12 m, where a series of spurs sloped gradually to 35+ m. On one side of the vertical terrace there was a 4-m diameter *Diploastrea* sp. colony. The base of the terrace and the areas within the wide grooves between the terraces (from 8 to 15 m) were dominated by large massive *Porites* sp. (1-2-m diameter and up to 1 m tall), and ALCC was 30-40%. Dead poritid framework and the surrounding substrate had been colonized by *Pocillopora* spp., digitate acroporids, *Goniopora* sp., and encrusting corals. The poritid colonies were less common below 15 m, and ALCC also declined to 20% below 15 m; ALCC was only 15% at 30 m. The deeper areas had lower relief (30-60 cm), with patches of hardground and sand interspersed with coral. The coral community was dominated by large, encrusting *Leptastrea* sp., as well as *Montipora* spp., *Isopora* sp., numerous branching *Stylophora* sp. and *Pocillopora* spp., and the occasional *Porites* sp. colony. Approximately 10-20% of the acroporids and pocilloporids had partial mortality and recent tissue loss from snail predation. Throughout the reef there were numerous, small (5-10 cm) stylophorids, as well as some acroporids and pocilloporids, that had been eaten by *Culcita* sp. seastars.

*TOVA39*. “Fingers.” This was a leeward, fringing forereef. The coast was a near vertical, uplifted reef that dropped vertically to a sand flat at 10-12 m. The sand flat had some large boulders that had tumbled into the water. About 50 m offshore there was a terrace that sloped from the sand gradually to 8 m depth. This was low-relief, scoured hardground with low cover of mostly encrusting corals (e.g., *Isopora* sp., *Montipora* sp., some digitate acroporids, and low-relief pocilloporids), large patches of turf with damselfish lawns, and large areas of relatively flat, CCA-encrusted rock. The shallow areas had 5-10% cover of soft corals. The terrace dropped near vertically on the sides and seaward edge to 15-18 m. A second series of large, low-relief boulders (10-15 m wide and up to 8 m tall) extended from 15-20 m to 8 m. There were also some spurs with 1-2-m deep, narrow channels that extended seaward, sloping gradually to the sand apron. In deeper waters there were some small sections of framework (up to 1 m above the surrounding substrate). The entire area had very low ALCC. There were some patches of coral on the spurs between 15 and 23 m (ALCC= 20%), interspersed with large areas that were devoid of corals. The vertical sides of the channels from 10 to 15 m had been partially colonized by sheets of *Pachyseris* sp., *Leptoseris* sp., and *Merulina* sp., but most other areas had low ALCC, 30-50% cover of CCA, and up to 50% turf algal cover. At 25-30 m there were some extensive patches of hydroids. Throughout the reef there were many small, dead stylophorids, pocilloporids, and acroporids, and about 20% of the *Isopora* sp., digitate acroporids, and pocilloporids had both old and recent mortality, with most of the latter presumably due to snails. Some snails were found preying on *Montipora* sp. colonies, and this site had the highest density of snails observed to date. One, several, and three pineapple cucumbers, giant clams, and *Culcita* sp. seastars were observed, respectively.

*TOVA40*. This lagoonal fringing reef sloped very gradually from 15 to 25 m before dropping more steeply to a soft-bottom community with a poritid framework. Massive poritids (0.5-2 m in diameter by 1 m height) were the dominant corals, with cover ranging from 20 to 40%. About 40% of the coral colonies had partial mortality, little of which was recent. In most cases, other massive and branching corals had colonized exposed skeletal surfaces. Scattered throughout the reef were large table acroporids and erect, thin-branched, and spindly staghorn acroporid colonies (some 1-2 m wide and 1 m tall). The second most abundant coral was *P. lobata*, with colonies up to 1 m in diameter and many with partial tissue loss. A high diversity of other corals was documented, including 1) massive and branching *Galaxea* spp., 2) *Favia* spp., 3) *Goniastrea* sp., 4) *Platygyra* sp., 5) *Coscinarea* sp., 6) *Astreopora* sp., 7) *P. rugosa* and other *Pachyseris* spp. plates, 8) *Merulina* spp., 9) *Leptoseris* sp., 10) bottlebrush acroporids, 11) *Pocillopora damicornis*, 12) *Seriatopora* spp., and 13) *Stylophora* sp., amongst numerous others. There were also 1) several large, round sponges, 2) branching sponges, and 3) a very common, grey, encrusting sponge with thin, upright branches that was overgrowing corals. The sediment in places had patches of cyanobacteria. Three species of sea cucumber were moderately common (pinkfish, an unknown spotted sp., and another *Bohadschia* sp.). One COTS was found to have recently eaten a large table acroporid and one other COTS was seen under a massive coral. Corallivorous snails (e.g., *Drupella* sp.) were not seen.

*TOVA41*. This leeward forereef was south of the channel. The topside cliffs dropped vertically to a sand flat at 5-8 m. From the sand flat, very low-relief spurs sloped very gradually to deeper waters. The spurs were 1-3 m wide and were characterized by narrow sand channels and up to 1 m of relief (20-40 cm of micro-relief on the reef itself). The spurs were hardground, with small corals, patches of sand, CCA, and very little turf algae except where damselfish lawns were present. At 15-18 m there was a minor build-up, with some spurs that were 2 m above the surrounding sand channels. The slope increased slightly below 20 m, continuing to 35+ m. There was no rubble in the sand flats, and very few large corals were observed on this reef. In shallow waters (5-10 m), the reef was dominated by *Pocillopora* spp. and *Acropora* spp. (up to 20% ALCC), and there were large areas of scoured hardground with CCA and some turf. From 10 to 15 m, the reef was dominated by 1) digitate acroporids, 2) *Pocillopora eydouxi* and *P. verrucosa*, 3) *Goniastrea retiformis* and *G. edwardsi*, 4) *Montipora* spp. crusts, 5) small *P. lobata* colonies, and 6) small *Montastrea* sp. colonies; there were lower numbers of *Astreopora* sp., *Pavona* sp. crusts, and other species. Below 20 m there were large piles of fungids amidst 1) massive and plating *Goniastrea* sp., 2) acroporids (mostly digitate though with some small tables), 3) *F. stelligera*, 4) *Mycedium* sp., 5) very small *Porites* spp., 6) numerous, encrusting *Leptastrea* sp., and 7) *Echinophyllia* sp. At 30 m the reef was dominated by *G. retiformis*, with lower numbers of acroporids, pocilloporids, favids, leptastreids, cyphastreids, montiporids, echinoporids, and *Pavona* sp. crusts. Staghorn corals were notably absent, as were sea cucumbers and large giant clams. Between 2 and 5% of the pocilloporids and acroporids had been affected by snail predation. Patches of *Halimeda* sp. occurred at the base of the reef and on vertical surfaces (up to 5% cover), and tufts of *Cladophora* sp. covered up to 5% of the benthos in deeper waters.

*TOVA42*. “Japanese Coral Gardens” (snorkel only). This was a large area running from shore towards three small, offshore islands. Closest to the islands was a flat hardground that ran from 0.5 m to 8-10 m before turning into sand. The slope was gentle, becoming steeper below 10 m. At depths of 1-3 m, there were numerous *P. damicornis* bommies that were 0.5-1 m tall and up to 20 m long; in addition to *P. damicornis*, there were also some staghorn corals and table acroporids. The corals were all about 30-50 cm above a hard bottom with primarily the ends of the branches (top 10-30 cm) alive; dense turf algae surrounded the corals’ bases/interiors. Also, many of the acroporids had dead spots and chimneys, with tufts of turf algae and cyanobacteria. Throughout these bommies there were hundreds of damselfish, and the turf was predominantly damselfish algal lawns. There were also numerous coral heads, including a few *P. lobata* bommies, as well as colonies of *P. rus*, *Favia* sp., *Astreopora* sp., *Goniastrea* sp., and branching *Millepora* sp. (some were 1 x 1 m). Most of the small coral heads and bommies that were not formed by *P. damicornis* were dead and covered in dense turf algae, erect red coralline algae, and/or cyanobacteria. In the sandy areas surrounding the bommies there were numerous sea cucumbers (at least three species), hundreds of *Oreaster* sp. seastars, and a few blue *Linkia* sp. and pink seastars. There were also more than 40 *Culcita* sp. seastars amongst the coral bommies and on the surrounding rubble. Throughout the *P. damicornis*-dominated areas, there were numerous, small, recently eaten coral heads (up to 20-cm diameters of white skeleton) with a lone *Culcita* sp. nearby. One bommie in the center of the patch (near the second island) was characterized by much more mortality and 15 COTS. Close to shore there were dense seagrass beds (*Thalassia* sp.), with some cyanobacteria, seastars, and sea cucumbers amidst them. On the slope, all of the coral heads were largely dead and covered in dense turf algae and erect red coralline algae. At the opposite end (south) of the islands at 5-10 m depth, there were patches of bottlebrush *Acropora* spp., *P. rugosa*, and a few other medium- to large-sized coral colonies at the margin between the hardground area and the sand flat.

*TOVA43*. The shallows of this lagoonal fringing reef, which was adjacent to the channel, had a reef terrace at 5-8 m. This sloped near vertically to 6-15 m, then continued to deeper waters at a 45-degree angle. The terrace and spurs in the shallows had very high relief (2-4 m), with up to 1 m of micro-relief on the tops of the spurs. This decreased with depth to 1-2 m of relief. The shallows were dominated by branching acroporids, pocilloporids, branching *Hydnophora* sp., *F. stelligera*, thick-branched *Isopora* sp., *Coscinarea* sp., and *Goniastrea* sp., many of which had damselfish algal lawns. The vertical surfaces and underhangs had many species of plating corals, including 1) two *Leptoseris* spp., 2) *Pavona maldivensis*, 3) *Pachyseris* sp., 4) *Mycedium* sp., and 5) *Echinophyllia* sp., amongst others. On the spurs, massive and plating corals dominated, especially encrusting/plating *Goniastrea* spp., *Isopora* sp., *Coscinarea* sp., and others. At 20 m there were very large *Lobophyllia* sp. colonies (2-4-m wide), as well as 1) large *Favites* sp., 2) *Porites* spp., 3) *Goniastrea* sp., 4) *Echinophyllia* sp., 5) *Montipora* spp., 6) small, digitate and staghorn acroporids, 7) some tabular acroporids, and 8) medium-sized pocilloporids. At 30 m and continuing to 40 m, there were large, overlapping sheets of *P. rus*, *Leptoseris* sp., and *Echinophyllia* sp. ALCC ranged from 10 to 15% in places to 20-25% in others, and there were large patches of monospecific assemblages covering 2-5 m at 60-80% cover. Throughout the reef and especially at the base of the shallow vertical drop, there were large piles of rubble, thick staghorn branches, and numerous dead, overturned, broken plates of coral.

*TOVA44*. At this lagoonal fringing reef, there was a sand flat close to shore, then a shallow reef platform that extended out to 100 m. This platform was 2-5 m deep with a scoured hardground near shore and very high cover of the following near the edge of the drop: 1) digitate, staghorn, and thick, tabular *Acropora* spp. (often with multiple canopy layers), 2) branching *Hydnophora* sp., 3) *Pocillopora* spp., 4) *Isopora* sp., 5) *Goniastrea* sp., and 6) *F. stelligera*, amongst other species. The platform dropped vertically to 8-12 m, then sloped steeply to 23-28 m, ending in sand. The vertical surfaces were covered in large, overlapping plates, including *Pachyseris* sp., *Leptoseris* sp., *Goniastrea* sp., *Echinophyllia* sp., *Oxypora* sp., *Echinopora* sp., and *Mycedium* sp., amongst others. There were also encrusting *Leptastrea* sp. and *Coscinarea* sp. colonies. At the base of this platform and running down the slope, there were large piles of broken, dead plates, dead branches, and rubble. The slope’s ALCC was 20-30%, and the assemblage consisted of table acroporids, high numbers of *G. retiformis*, *Stylophora* sp., *F. stelligera*, *Echinopora* sp., *Merulina* sp., *P. rus*, *Turbinaria* sp. (often growing as a series of overlapping shingles), and other species at lower abundances. Approximately 20-30% of the table acroporids were dead (in growth position), and 5% had large, white lesions. Few predatory snails were observed, and although only one *Culcita* sp. and one COTS were seen, there were numerous, small, dead acroporids and pocilloporids. There were numerous sea cucumbers, and high cover of CCA characterized all depths. Soft corals were mostly limited to the shallows.

*TOVA45*. This was a fringing reef on the leeward side of a small island in the southwest of the archipelago. The reef sloped gently from 3-5 m, where relief was only 30-60 cm. There was some framework at 6-8 m (up to 1 m of relief), where multiple canopy layers could be observed; then, the reef sloped slightly more steeply to 20 m where the reef ended in sand. There were scattered, thick-branched isoporids in the sand, followed by a second reef system (50-100 m seaward) that started at 35 m and sloped to the depths. The shallows (< 5 m) had very low ALCC, with scattered corals present and 10-20 cm of relief. ALCC was much higher at 5 m (30-50%), but the assemblage was dominated by small corals (up to 30-40 cm in height): 1) digitate, small, thick-branched, tabular corals and 2) small- to medium-sized favids, amongst other hard coral species. At the edge of the slope to about 12 m, there were patches of coral with small, thick-branched staghorn corals and small thickets (up to 3 m across) intermixed with other acroporids, pocilloporids, favids, *Coscinarea* sp., *Hydnophora* sp., plates of *Montipora* spp., and other species. ALCC declined to 20% at 15 m and 10-15% at 30 m. This area had more rubble, as well as scattered areas with high coral cover intermixed with those of low ALCC. There were numerous massive coral species, including 1) medium-sized *F. stelligera* colonies, 2) *Leptoria* sp., 3) small poritids, 4) *Favites* sp. and *Goniastrea* sp., 5) many *Cyphastrea* sp. colonies, and 6) plates of *Mycedium* sp. There were also several stands of staghorn coral. The reef had patches of up to 30% *Halimeda* sp. cover, small (1-2 m), dense patches of *Caulerpa* sp., and about 10% and 20% cyanobacteria and turf algal cover, respectively. Finally, there were moderately high numbers of sea cucumbers.

*TOVA46*. This barrier reef on the leeward side of the southwestern end of Vava’u’s reef system was gently sloping and characterized by 1) wide spurs, 2) wide sand patches and channels, and 3) little rubble. The shallow areas (8-12 m) had up to 1.5 m of relief, but relatively low ALCC; the assemblage was dominated by favids, small acroporids, pocilloporids, and stylophorids. At 12-15 m there was very little relief; it was largely scoured with little coral and 40-50% soft coral cover. At 15-25 m the spurs were 2-3 m above the surrounding sand, with 1) large patches of staghorn corals (most of which were partially dead), 2) dense turf algal patches (likely damselfish gardens), 3) soft corals, 4) scattered, large table acroporids (up to 2 m in diameter and 10% dead), 5) *Isopora* sp., 6) *Goniastrea* sp., 7) *Montipora* spp. plates, 8) *Stylophora* sp. colonies (most with partial mortality and predatory snails), 9) *F. stelligera*, 10) *Coscinarea* sp., and 11) *Porites* sp. colonies (1-2 m in diameter). One very large *Porites* sp. colony (3 x 2 m) was observed at the edge of one spur in the sand (15 m), as was one very large (2 m in diameter) *Montipora* sp. plate. The reef had 10-15% cover of *Halimeda* sp., good CCA cover (30%) throughout all depths, and 20% cover of turf algae (higher in damselfish algal lawn areas). Low numbers of sea cucumbers and three *Culcita* sp. seastars were documented.

*TOVA47*. The shallows (3-8 m) of this fringing reef on the leeward side of an island had high ALCC (50-80%), and the assemblage consisted of branching acroporids, staghorn acroporids, digitate acroporids, table acroporids, thick-branched acroporids, pocilloporids, *Hydnophora* sp., favids, *Coscinarea* sp., and many other species. Soft coral cover was 20-30%. Most corals were in excellent condition, and few predatory snails were seen; only 5% of the acroporids were dead. There was a coral-based build-up up to 1.5 m, but there were no real grooves or sand channels. On the steep slope, ALCC declined to 20-30%, and there were numerous overturned and broken corals; despite this, the slope had an interesting and diverse assemblage of corals, including *Echinophyllia* sp., *Astreopora* sp., various acroporids, pocilloporids, *Coscinarea* sp., numerous favids, *Galaxea* sp., *Montastrea* sp., *Montipora* sp., *Mycedium* sp., and *Acanthastrea* sp., amongst others. The shallow slope had some large stands of branching *Porites* spp.; in several deeper locations there were 2-5-m assemblages of bottlebrush acroporids. Table acroporids below 15 m were up to 2 m wide, and about 10% of the colonies were dead. The reef ended at 30-32 m in sand. At the base of the reef there were small thickets of staghorn coral. There was little macroalgae (2% cover). Turf algae and CCA cover was 30%, and cyanobacteria comprised 2-5% of the benthos. Many sea cucumbers were present, as were several large giant clams. Three *Culcita* sp. seastars were seen, and 1-3% of corals were covered with predatory snails.

*TOVA48-50*: No site descriptions were produced for these three sites.

*TOVA51*. Northwest end of Treasure Island. No site description was produced for this site.

*TOVA52*. Coral pinnacles off Ofu Reef South. No site description was produced for this site.

*TOVA53*. This fringing reef off the southwestern end of “Treasure Island” had a wide shallow area at 3-5 m with very high cover (ALCC=60-80%) of 1) branching corals (especially digitate, thick, table, and staghorn acroporids), 2) *Montipora* sp., 3) *Pocillopora* spp., 4) *F. stelligera*, and 5) *Goniastrea* sp. colonies; the latter were covered with damselfish lawns in some areas. At 10 m the slope became mostly sandy, with large areas of rubble interspersed amongst coral patches. There were small table acroporids, *Montastrea* sp., *Favia* sp., and *Mycedium* sp., amongst others. ALCC increased to 10-15% at 15 m, then dropped to 5-10% at 20 m and 1-2% at 30 m, before ending in sand. The slope had numerous table acroporids, *Mycedium* sp., *Montipora* spp. plates, *Merulina* sp., *Favites* sp., *Leptastrea* sp., and small patches of *P. rugosa*. Four, two, and eight *Culcita* sp. seastars, trumpet tritons, and sea cucumbers (most at 25 m), respectively, were seen. Patches of *Halimeda* sp. (especially at deeper depths) covered 20-30% of the benthos.

**Niuatoputapu Volcano**

*TONI54.* This windward, fringing forereef at the southern tip of the island sloped very gradually from 4-5 m to 25 m. Small, discontinuous spurs were surrounded by extensive low- relief areas (up to 50 cm) where rubble with small corals were common. At 10 m there was a larger spur with massive *Astreopora* sp., *Montipora* spp., *Porites* spp., and table acroporids (ALCC=30-50%). ALCC remained fairly high at 15 m (30-50%), and the assemblage was dominated by digitate and table acroporids, *Montipora* spp. crusts, *Montastrea* sp., *Echinopora* sp., and various staghorn coral species. Slightly deeper there were more *Porites* spp., *Goniastrea* sp., *Coscinarea* sp. (which formed large columns), stout-branched *Isopora* sp., and foliaceous *Turbinaria* sp., amongst many others. The reef had large amounts of *Halimeda* sp. (30% cover), and 2-5% of the benthos was covered with damselfish algal lawns. About 30% of the corals were dead, and there was a large, dead stand of thick-branched staghorn corals at 15 m. There were numerous diseased pocilloporids.

*TONI55.* This leeward forereef off the northwestern tip of the island’s reef system possessed an extensive, shallow-water platform that ranged from 8 to 20 m depth. The reef spurs extended for hundreds of meters, sloping very gently. These were surrounded by sand patches, some with large boulders and others with little rubble. There were very few acroporids throughout the reef system. ALCC was 10-40%, and the scleractinian assemblage was dominated by large, mounding *Astreopora* sp. colonies, encrusting and plating *Montipora* spp., small- to medium-sized poritids (most of low relief; up to 30-40 cm tall and less than 1 m in diameter), numerous small- to medium-sized pocilloporids, *Montastrea* sp., and patches with medium to large *Turbinaria* spp. colonies; some areas had 6-8 foliaceous *Turbinaria* spp. colonies over 1 m in diameter. Poritids were dominant below 15 m, though they were much less common in the shallows. *Turbinaria* spp. included the yellow/green *T. mesenterina* and *T. stellata*. There was also a very high abundance of blade-like *Millepora* sp., with colonies from 30 to 60 cm in diameter and up to 40 cm tall; this was the first survey site to have been colonized by this species to any great extent. The relief on the spurs was moderately low; although some coral heads were up to 80 cm tall, most were only 30-60 cm. About 20-30% of the pocilloporid colonies had recent mortality, as well as old dead patches caused by disease. Predatory snails were seen, albeit at low densities. A few *Culcita* sp. seastars were observed in the vicinity of recently dead corals, but starfish were generally rare at the site. There was moderately good cover of CCA, sparse turf algae, and little macroalgae (a few tufts of *Cladophora* sp.). There was about 5% cover of *Halimeda* sp. and little cyanobacteria (1-2% cover in places). Few sea cucumbers were seen, most of which were uniformly black.

*TONI56*. This fringing reef on the west coast of the island was characterized by relatively low-relief mounds surrounded by sand and rubble. There was a long ridge (2-3 m wide and 2 m above the surrounding rubble/sand) that sloped gradually from 8 to 15 m. The reef had moderately high ALCC: 30-50% at 12-15 m before dropping to 20-40% at 20 m. The scleractinian assemblage was dominated by 50-100-cm diameter *Porites* spp., *Astreopora* sp., *Montipora* spp., *Turbinaria* spp., and *Pocillopora* spp. There were few acroporids, though there were some staghorn corals on the ridge and a large stand of *Isopora* sp. at the deeper end of the ridge. Small patches of *Halimeda* sp. occurred throughout the reef. Turf algal cover ranged from 30 to 50% and was densest in damselfish lawns. There were 1) very high numbers of white *Linkia* sp. seastars, 2) numerous, large giant clams, and 3) dozens of spider conch. Many *Astreopora* sp. colonies had small, dead areas that formed chimneys with tufts of red algae. Approximately 10-20% of the pocilloporids were diseased.

*TONI57*. This was a barrier reef along the north coast (west of the channel). Large spurs extended from 3-4 m to 20 m, ending in sand. In the shallows there were very narrow channels (up to 3-4 m deep). These became wider and lower relief in deeper waters. The tops of the spurs had 10-30 cm of micro-relief, while the sides consisted of a cemented branching and massive coral framework with 40-80 cm of relief. This area had small table acroporids, pocilloporids, small favids, *Montastrea* sp., and *Montipora* spp. At the base of the spurs there were more digitate and tabular acroporids, some larger plates of *Turbinaria* spp., patches of staghorn coral, *Merulina* sp., *Leptastrea* sp., and other stony corals. At 20-30 m the spurs and mounds were characterized by much lower relief, with scattered coral bommies and rocks up to 1 m in diameter intermixed with some larger *P. lobata* colonies and table acroporids. The reef appeared to have suffered significant damage from a tsunami; extensive patches of rubble were evident, but most had been cemented together with thick CCA crusts. There was considerable recolonization, mostly thin, plating sheets of *Montipora* spp. at 5-30% cover. Deeper areas had scattered, larger table acroporids that were undamaged. Many of the pocilloporid colonies were diseased. There were large patches of cyanobacteria. Few predatory snails and no *Culcita* sp. seastars were seen.

*TONI58*. This submerged forereef off the northwest of the island had considerable areas of sand and was mostly flat (1-2-m tall spurs with little rugosity [30-50 cm]); ALCC was 5-10%. The reef sloped gradually from 5 m to 15-18 m, ending in a sand apron with coral bommies. The sand flat continued to slope very gradually to 25 m. Extensive areas of dead coral pillars (either *Isopora* sp. spires or *P. clavus* colonies) were present at 15-18 m. The pillars were easily broken and had multiple, thick layers of CCA. This area also had 1) scattered, living colonies of *Isopora* spp., 2) *Pocillopora* spp., 3) *Goniastrea* spp., 4) small, finely branched table acroporids, 5) small favids, and 6) some 60-100-cm diameter *Turbinaria* spp. colonies. Other spurs from 15-20 m depth had been colonized by thin, plating *Montipora* sp. and other species, with more table acroporids and 0.5-1-m diameter *Porites* spp. colonies at 20 m. Throughout the sandy areas there were numerous coral bommies up to 3 m tall and 2-3 m wide. These were all *P. lobata* framework; many were mushroom-shaped and had living *P. lobata* on their caps, with little live coral tissue on the sides. Often, parts of the original poritid colonies had died, and other corals (especially branching acroporids) had colonized the bommies. There were also some lower relief *Porites* sp. domes (0.5-1 m wide and 30-100 cm tall).

*TONI59*. The last reef site surveyed was a barrier reef off the north coast. The shallow reef (3-8 m) had a gradual slope, with high cover of small pocilloporids and acroporids. Deeper there were 1-2-m tall spurs surrounded by sand and rubble. The framework had 30-60 cm of relief. At 8-15 m, ALCC was 30-40%, and the assemblage was dominated by numerous, very large (1-2 m in diameter), foliaceous plates of *Turbinaria* sp. From 16 to 20 m, the tops of the spurs were dominated by table acroporids (most of which were less than 50 cm in diameter), and the following were seen on the sides of the spurs: 1) small, thin, yellow/green *Montipora* sp. plates, 2) small *Porites* spp. colonies, and 3) *Pocillopora* spp. colonies. At 20-25 m there were scattered coral bommies in the sand, most of which were *Porites* framework. One very large *Porites* sp. colony (10-m diameter and 2-3 m tall) that had lost ~80% of its tissue was seen; the living poritid tissue consisted of mostly 5-15-cm diameter patches, as well as a few larger patches. Parts of the coral colonies had been smothered by damselfish algal lawns. Other areas were colonized to varying degrees by acroporids, pocilloporids, and many other species. CCA cover was high (40-50% in the shallows, decreasing to 20-30% at 25 m). Turf algal cover ranged from 20 to 50% and was highest in areas with damselfish lawns. No predatory snails, COTS, or *Culcita* sp. seastars were seen, but there were large numbers of diseased pocilloporids.
