# Supplementary material for: Biomarker profiling in reef corals of Tonga’s Ha’apai and Vava’u archipelagos
Source: PLoS One. 2017 Nov 1;12(11):e0185857. doi: 10.1371/journal.pone.0185857 (PMC5665425; doi:10.1371/journal.pone.0185857)
Supplement: S1 Table — The values below the environmental parameters (EP; top row) represent the number of categorical groupings. X2 tests and 1-way ANOVAs were used to analyze the frequency (freq.) and molecular+physiological data, respectively. Comparisons that were statistically significant at the Bonferroni-adjusted α levels of 0.013, 0.013, 0.013, 0.004, and 0.013 for the host freq. X2 tests, outlier freq. X2 tests, polyp expansion freq. X2 tests, molecular-scale response variable (MSRV) ANOVAs, and multivariate ANOVAs (MANOVA; Wilks’ lambda was calculated.), respectively, are highlighted in green, and marginally significant p-values have been highlighted in yellow. The P. verrucosa (n = 1) and P. meandrina (n = 1) samples were excluded from the analysis. ALCC = average live coral cover. Sym = Symbiodinium. NS = not statistically significant. NA = not applicable. (DOCX) [file pone.0185857.s003.docx]

**S1 table. Univariate statistical analysis of the Tonga dataset-I: host species analyzed together**. The values below the environmental parameters (EP; top row) represent the number of categorical groupings. *X*^2^ tests and 1-way ANOVAs were used to analyze the frequency (freq.) and molecular+physiological data, respectively. Comparisons that were statistically significant at the Bonferroni-adjusted α levels of 0.013, 0.013, 0.013, 0.004, and 0.013 for the host freq. *X*^2^ tests, outlier freq. *X*^2^ tests, polyp expansion (expan.) freq. *X*^2^ tests, molecular-scale response variable (MSRV) ANOVAs, and multivariate ANOVAs (MANOVA; Wilks’ lambda was calculated.), respectively, are highlighted in green, and marginally significant *p*-values have been highlighted in yellow. The *P. verrucosa* (n=1) and *P. meandrina* (n=1) samples were excluded from the analysis. ALCC= average live coral cover. Sym= *Symbiodinium*. NS=not statistically significant. NA=not applicable.

^a^log-transformed data. ^b^square root-transformed data. ^c^rank-transformed data. ^d^*z*-scores. ^e^see Fig 4.

| **EP/**  **MPRV** | archi-pelago (n=2) | site (n=21) | exposure (n=3) | reef zone (n=3) | reef type (n=3) | date (n=16) | time (n=3) | depth (n=6) | temp. (n=5) | salinity (n=4) | ALCC  (n=5) | host (n=2) | color (n=4) | Sym assemblage (n=3) |
| --- | --- | --- | --- | --- | --- | --- | --- | --- | --- | --- | --- | --- | --- | --- |
| host freq. | <0.01 | 0.001 | NS | NS | NS | <0.001 | <0.01 | NS | NS | NS | NS | NA | NS | NS |
| polyp expan.  freq. | NS | <0.001 | NS | <0.013 | NS | <0.001 | NS | NS | <0.001 | NS | NS | <0.001 | <0.004 | NS |
| outlier freq. | NS | NS | NS | NS | NS | NS | NS | NS | <0.013 | NS | NS | NS | NS | NS |
| max. length^a^ | NS | <0.001 | NS | NS | NS | <0.001 | NS | NS | <0.004 | NS | <0.004 | NS | NS | NS |
| planar SA^a^ | NS | <0.001 | NS | NS | NS | <0.001 | NS | NS | 0.006 | 0.007 | NS | NS | NS | NS |
| Sym GCP^b^ | NS | NS | NS | NS | NS | NS | NS | NS | NS | NS | NS | NS | NS | NS |
| RNA/DNA^a^ | NS | NS | NS | NS | NS | NS | NS | NS | NS | NS | NS | NS | NS | NS |
| Sym *rbcL*^c^ | NS | NS | NS | NS | NS | NS | NS | NS | NS | 0.009 | NS | NS | NS | NS |
| Sym *zifl1l*^c^ | NS | NS | NS | NS | NS | NS | <0.001^e^ | NS | NS | NS | NS | NS | NS | NS |
| Sym *hsp90*^c^ | NS | NS | NS | NS | NS | NS | NS | NS | NS | NS | NS | NS | NS | NS |
| Sym *ubiq-lig*^c^ | NS | NS | NS | NS | NS | NS | NS | NS | NS | NS | NS | NS | NS | NS |
| host *ca*^c^ | NS | NS | NS | NS | NS | NS | NS | NS | NS | NS | NS | NS | NS | NS |
| host *lectin*^a^ | NS | NS | NS | NS | NS | NS | NS | NS | NS | NS | NS | NS | NS | NS |
| host *cu-zn-*  *sod*^c^ | NS | NS | NS | NS | NS | NS | NS | NS | NS | NS | NS | NS | NS | NS |
| host *gfp-cp*^a^ | NS | NS | NS | NS | NS | NS | NS | NS | NS | NS | NS | NS | NS | NS |
| multivariate mean^d^ | NS | NS | NS | NS | NS | NS | <0.004^e^ | NS | NS | NS | NS | NS | NS | NS |
