# Supplementary material for: Biomarker profiling in reef corals of Tonga’s Ha’apai and Vava’u archipelagos
Source: PLoS One. 2017 Nov 1;12(11):e0185857. doi: 10.1371/journal.pone.0185857 (PMC5665425; doi:10.1371/journal.pone.0185857)
Supplement: S2 Table — The values below the environmental parameters (EP; top row) represent the number of categorical groupings. X2 tests and 1-way ANOVAs were used to analyze the frequency (freq.) and molecular+physiological data, respectively. Comparisons that were statistically significant at the Bonferroni-adjusted α levels of 0.013, 0.013, 0.004, and 0.013 for the outlier freq. X2 tests, polyp expansion (expan.) freq. X2 tests, molecular physiological response variable (MPRV) ANOVAs, and multivariate ANOVAs (MANOVA; Wilks’ lambda was calculated.), respectively, are highlighted in green, and marginally significant p-values have been highlighted in yellow. ALCC = average live coral cover. Sym = Symbiodinium. NS = not statistically significant. (DOCX) [file pone.0185857.s004.docx]

**S2 table. Univariate statistical analysis of the Tonga dataset-II: host species analyzed separately**. The values below the environmental parameters (EP; top row) represent the number of categorical groupings. *X*^2^ tests and 1-way ANOVAs were used to analyze the frequency (freq.) and molecular+physiological data, respectively. Comparisons that were statistically significant at the Bonferroni-adjusted α levels of 0.013, 0.013, 0.004, and 0.013 for the outlier freq. *X*^2^ tests, polyp expansion (expan.) freq. *X*^2^ tests, molecular physiological response variable (MPRV) ANOVAs, and multivariate ANOVAs (MANOVA; Wilks’ lambda was calculated.), respectively, are highlighted in green, and marginally significant *p*-values have been highlighted in yellow. ALCC= average live coral cover. Sym=*Symbiodinium*. NS=not statistically significant.

| **EP/**  **MPRV** | archi-pelago (n=2) | site (n=21) | exposure (n=3) | reef zone (n=3) | reef type (n=3) | date (n=16) | time (n=3) | depth (n=6) | temp. (n=5) | salin-ity (n=4) | ALCC  (n=5) | color (n=4) | Sym assemblage (n=3) |
| --- | --- | --- | --- | --- | --- | --- | --- | --- | --- | --- | --- | --- | --- |
| ***Pocillopora damicornis*** | |  |  |  |  |  |  |  |  |  |  |  |  |
| polyp expan. freq. | NS | NS | NS | NS | NS | *p*=0.013 | NS | NS | NS | NS | NS | NS | NS |
| outlier freq. | NS | NS | NS | NS | NS | NS | NS | NS | NS | NS | NS | NS | NS |
| max. length^a^ | NS | NS | NS | NS | NS | NS | NS | NS | NS | NS | NS | NS | NS |
| planar SA^a^ | NS | NS | NS | NS | NS | NS | NS | NS | NS | NS | NS | NS | NS |
| Sym GCP^b^ | NS | NS | NS | NS | NS | NS | NS | NS | NS | NS | NS | NS | NS |
| RNA/DNA^a^ | NS | NS | NS | NS | NS | NS | NS | NS | NS | NS | NS | NS | NS |
| Sym *rbcL*^c^ | NS | NS | NS | NS | NS | NS | NS | NS | NS | NS | NS | NS | NS |
| Sym *zifl1l*^c^ | NS | NS | NS | NS | NS | NS | NS | NS | NS | NS | NS | NS | NS |
| Sym *hsp90*^c^ | NS | NS | NS | NS | NS | NS | NS | NS | NS | NS | NS | NS | NS |
| Sym *ubiq-lig*^c^ | NS | NS | NS | NS | NS | NS | NS | NS | NS | NS | NS | NS | NS |
| host *ca*^c^ | NS | NS | NS | NS | NS | NS | NS | NS | NS | NS | NS | NS | NS |
| host *lectin*^a^ | NS | NS | NS | NS | NS | NS | NS | NS | NS | NS | NS | NS | NS |
| host *cu-zn-sod*^c^ | NS | NS | NS | NS | NS | NS | NS | NS | NS | NS | NS | NS | NS |
| host *gfp-cp*^a^ | NS | NS | NS | NS | NS | NS | NS | NS | NS | NS | NS | NS | NS |
| multivariate mean^d^ | NS | NS | NS | NS | NS | NS | NS | NS | <0.01 | NS | <0.01 | NS | NS |
| ***Pocillopora acuta*** | |  |  |  |  |  |  |  |  |  |  |  |  |
| polyp expan. freq. | NS | NS | NS | NS | NS | *p*=0.013 | NS | NS | <0.01 | NS | NS | NS | NS |
| outlier freq. | NS | NS | NS | NS | NS | NS | NS | NS | NS | NS | NS | NS | NS |
| max. length^a^ | NS | <0.0001 | NS | NS | NS | <0.0001 | NS | NS | <0.0001 | NS | <0.004 | NS | NS |
| planar SA^a^ | NS | <0.0001 | NS | NS | NS | <0.0001 | NS | NS | <0.0001 | NS | <0.004 | NS | NS |
| Sym GCP^b^ | NS | NS | NS | <0.004 | NS | NS | NS | NS | NS | NS | NS | NS | NS |
| RNA/DNA^a^ | NS | NS | NS | NS | NS | NS | NS | NS | NS | NS | NS | NS | NS |
| Sym *rbcL*^c^ | NS | NS | NS | NS | NS | NS | NS | NS | NS | NS | NS | NS | NS |
| Sym *zifl1l*^c^ | NS | NS | NS | NS | NS | NS | <0.0001^e^ | NS | NS | NS | NS | NS | NS |
| Sym *hsp90*^c^ | NS | NS | NS | NS | NS | NS | NS | NS | NS | NS | NS | NS | NS |
| Sym *ubiq-lig*^c^ | NS | NS | NS | NS | NS | NS | NS | NS | NS | NS | NS | NS | NS |
| host *ca*^c^ | NS | NS | NS | NS | NS | NS | NS | NS | NS | NS | NS | NS | NS |
| host *lectin*^a^ | NS | NS | NS | NS | NS | NS | NS | NS | NS | NS | NS | NS | NS |
| host *cu-zn-sod*^c^ | NS | NS | NS | NS | NS | NS | NS | NS | NS | NS | NS | NS | NS |
| host *gfp-cp*^a^ | NS | NS | NS | NS | NS | NS | NS | NS | NS | NS | NS | NS | NS |
| multivariate mean^d^ | NS | NS | NS | NS | NS | NS | <0.01^e^ | NS | NS | NS | NS | NS | NS |

^a^log-transformed data. ^b^square root-transformed data. ^c^rank-transformed data. ^d^*z*-scores. ^e^see Fig 4.
